# Effects of sex and chronic cigarette smoke exposure on the mouse cecal microbiome

**DOI:** 10.1371/journal.pone.0230932

**Published:** 2020-04-06

**Authors:** Anthony Tam, Fernando Sergio Leitao Filho, Seung Won Ra, Julia Yang, Janice M. Leung, Andrew Churg, Joanne L. Wright, Don D. Sin

**Affiliations:** 1 Department of Medicine, Centre for Heart Lung Innovation, St. Paul’s Hospital, Vancouver, British Columbia, Canada; 2 Ulsan University Hospital, University of Ulsan College of Medicine, Ulsan, Korea; 3 Department of Pathology, University of British Columbia, Vancouver, British Columbia, Canada; Mayo Clinic Rochester, UNITED STATES

## Abstract

**Rationale:**

Chronic smoke exposure is associated with weight loss in patients with Chronic Obstructive Pulmonary Disease (COPD). However, the biological contribution of chronic smoking and sex on the cecal microbiome has not been previously investigated.

**Methods:**

Adult male, female and ovariectomized mice were exposed to air (control group) or smoke for six months using a standard nose-only smoke exposure system. DNA was extracted from the cecal content using the QIAGEN QIAamp® DNA Mini Kit. Droplet digital PCR was used to generate total 16S bacterial counts, followed by Illumina MiSeq® analysis to determine microbial community composition. The sequencing data were resolved into Amplicon Sequence Variants and analyzed with the use of QIIME2^®^. Alpha diversity measures (Richness, Shannon Index, Evenness and Faith’s Phylogenetic Diversity) and beta diversity (based on Bray-Curtis distances) were assessed and compared according to smoke exposure and sex.

**Results:**

The microbial community was different between male and female mice, while ovariectomy made the cecal microbiome similar to that of male mice. Chronic smoke exposure led to significant changes in the cecal microbial community in both male and female mice. The organism, *Alistipes*, was the most consistent bacteria identified at the genus level in the cecal content that was reduced with chronic cigarette exposure and its expression was positively related to the whole-body weight of these mice.

**Conclusion:**

Chronic smoke exposure is associated with changes in the cecal content microbiome; these changes may play a role in the weight changes that are observed in cigarette smokers.

## Introduction

Cigarette smoking is associated with weight loss but the mechanism for this is not well known [1–3]. The traditional paradigm is that tobacco contains nicotine, which reduces appetite and decreases food intake [4, 5], but other factors may also play a role. For instance, chronic smoke exposure promotes tissue hypoxia and mucosal inflammation in the gastrointestinal tract and also decreases intestinal barrier function [6]. The deleterious effects of smoking may hamper gut permeability and perturb the ability to absorb nutrients through the gut lining [7]. Cigarette smoking also predisposes to the development of inflammatory conditions such as ulcers, inflammatory bowel disease and cancer [6, 8], which further contribute to a dysfunctional gut environment.

It is widely accepted that a healthy gut microbiome is associated with adequate cellular metabolism and energy extraction from diet [9–11]. There is also a growing body of evidence that cigarette smoking may promote shifts in the gut microbial communities, leading to an imbalance between commensals and pathogenic bacteria (i.e., gut microbial dysbiosis) [12, 13], which may impact on weight. Consistent with this notion, the genera *Bifidobacteria* and *Lactococcus*, which are involved in energy metabolism from short-chain fatty acids, are decreased in the intestinal flora following smoke exposure [13–15]. In a previous study, Bäckhed and colleagues reported that germ-free (GF) mice bred in isolation and free from detectable bacteria, viruses or eukaryotic microbes showed significantly lower body weight compared with conventionally-raised mice [9]. Interestingly, the GF mice after receiving cecal contents from conventionally-raised mice had a 61% increase in their epididymal fat weight, supporting the importance of the gut microbiome in regulating body weight.

Moreover, the effects of sex and sex hormones on the gut microbiome in mice are increasingly being recognized [16–20]. Org and colleagues detected significant sex and hormonal (ovariectomy and gonadectomy) differences in the gut microbial community in C57BL/6J, C3H/HeJ and DBA/2J mice [20]. However, the impact of sex hormones on the cecal microbiome of mice after chronic smoke exposure has not been previously investigated. As women are more susceptible to adverse effects from cigarette smoking [21, 22], we sought to determine the role of sex and female sex hormones on the cecal microbiome in response to chronic cigarette smoke exposure.

## Materials and methods

The data reported in the present study were generated from mouse samples collected from a previous study where we examined the effect of sex-related differences on pulmonary function from chronic cigarette exposure [23]. In the current study, we report additional data focusing on the cecal microbiome and its contribution to changes in whole-body weight after chronic smoke exposure.

### Animals

Adult male, female and ovariectomized C57BL/6 female mice (12 weeks old) were obtained from Charles River (Montreal, PQ, Canada) [23]. In brief, surgical ovariectomy of female mice was performed at Charles River four weeks prior to cigarette smoke exposure. 1R1 and 2R4F research grade cigarettes were obtained from the University of Kentucky (Lexington, KY, USA).

### Smoke exposure

We studied 6 groups of mice (n = 10 per group): 1) control males (CM), 2) smoke-exposed males (SM), 3) control females (CF), 4) smoke-exposed females (SF), 5) control ovariectomized females (COF), and 6) smoke-exposed ovariectomized females (SOF). The smoke-exposed groups were consistently exposed to three cigarettes (one 1R1 and two 2R4F with the filters removed, or two 1R1 and one 2R4F with the filters removed on every other smoking day) for five days per week for six months. Two mice died in the smoke-exposed ovariectomized group from acute bronchospasm. All smoke exposures were conducted using our standard nose-only smoke exposure system [23–26].

### Sample processing

Immediately after the last smoke exposure, cecal content was placed into a 1.5 mL microcentrifuge tube and immediately frozen in dry ice and stored in -80°C. DNA extraction was performed using the Qiagen DNeasy & Stool Extraction Kit according to the manufacturer’s protocol (Qiagen, Hilden, Germany). Nanodrop (Thermo Fisher Scientific, Wilmington, USA) was performed on each sample to assess the quality and quantity of the DNA.

### Microbiome profiling

Details regarding the PCR amplification of the 16S rRNA gene V4 region were previously reported [27]. The cecal microbiome was determined by polymerase chain reaction (PCR) amplification of the 16S rRNA gene V4 region using the Illumina MiSeq^®^ platform (2 x 250 bp) [28]. All samples were sequenced at the University of British Columbia (UBC) Sequencing and Bioinformatics Consortium. Due to low quality of the reverse reads, only the forward reads were considered in this analysis. Sequencing reads were de-noised using DADA2 (Divisive Amplicon Denoising Algorithm) [29] and clustered into Amplicon Sequence Variants (ASV) [30] using QIIME2^®^ (Quantitative Insights into Microbial Ecology) [31]. Six extraction negative controls (in which no sample DNA was added during the DNA extraction steps) were used to identify potential contaminants in our study. S1 Fig provides the 16s RNA gene copies/μL for both cecal and extraction negative samples. ASVs, which were present in at least two controls and showed higher average relative abundance compared to cecal samples, were considered potential contaminants and thus removed from downstream analysis. S1 Table in the Online Data Supplement provides the taxonomic annotations for these ASVs. In addition, sequencing reads related to ASVs not classified at the phylum level, singletons, and low abundant taxa (total frequency less than ten times across all samples) were also removed from the final analysis. All cecal samples were rarified to the lowest number of reads observed across all samples (7,143 reads) during alpha and beta diversity analyses. The SILVA 16s rRNA gene reference database (v132, set NR99) was applied to assign bacterial taxonomic classifications [32]. Sequences are available via the National Center for Biotechnology Information Sequence Read Archive (accession number PRJNA589852).

### Statistical analysis

16S counts were analyzed using t-tests. The feature table (which contains the frequencies of each unique ASV in each sample in the dataset) and taxonomic annotations obtained from QIIME2^®^ were obtained and exported as a BIOM file [33] and analyzed in R (version 3.6.1, available http://www.R-project.org), using the Vegan (version 2.5–6) [34] and Phyloseq (version 1.30.0) [35] packages. Alpha diversity measures (Richness, Shannon Index, Pielou's Evenness Index and Faith's Phylogenetic Diversity—PD) [36] were expressed as median [interquartile range–IQR] and compared according to smoke exposure (control and smoke-exposed samples), sex (males, females, and ovariectomized females), and after stratification by smoke exposure and sex. These comparisons were performed using non-parametric methods (Wilcoxon rank-sum or Kruskal-Wallis tests as appropriate) [37]. Microbial communities (beta diversity) were also assessed based on Bray Curtis distances [37] and compared between groups of interest using principal coordinates analysis (PCoA) plots and Permutational Multivariate Analysis of Variance (PERMANOVA) using 1,000 permutations [31, 38]. Relative abundances (RA) of the most frequent phyla and genera (displayed as median [IQR]), were also obtained and compared using a similar approach as described for alpha diversity analyses. We used the Benjamini-Hochberg method to obtain adjusted p-values related to pairwise comparisons for all microbiome analyses (alpha diversity metrics, beta diversity and relative taxa abundances) [39]. To identify taxa features differentially expressed between control and smoke-exposed samples or across different groups, we also used the linear discriminant analysis (LDA) effect size (LEfSe) method [40]. LEfSe’s algorithm uses non-parametric tests (Kruskal–Wallis test followed by multiple Wilcoxon rank-sum tests) to adjust its results for multiple comparisons. The taxa features identified by LEfSe (LDA score > 3.5) were exported and visualized in R using the Yingtools2 package (version 0.0.0.90, available at https://github.com/ying14/yingtools2). We also used Prism 8 (GraphPad Software Inc. La Jolla California) for additional statistical analyses and graph generation. The level of statistical significance was set at p<0.05 (two-tailed) for all tests.

### Study approval

All procedures were previously approved by the University of British Columbia Animal Care Committee (A11-0149).

## Results

### Chronic smoke exposure reduced the ability to gain weight

**Fig 1:**
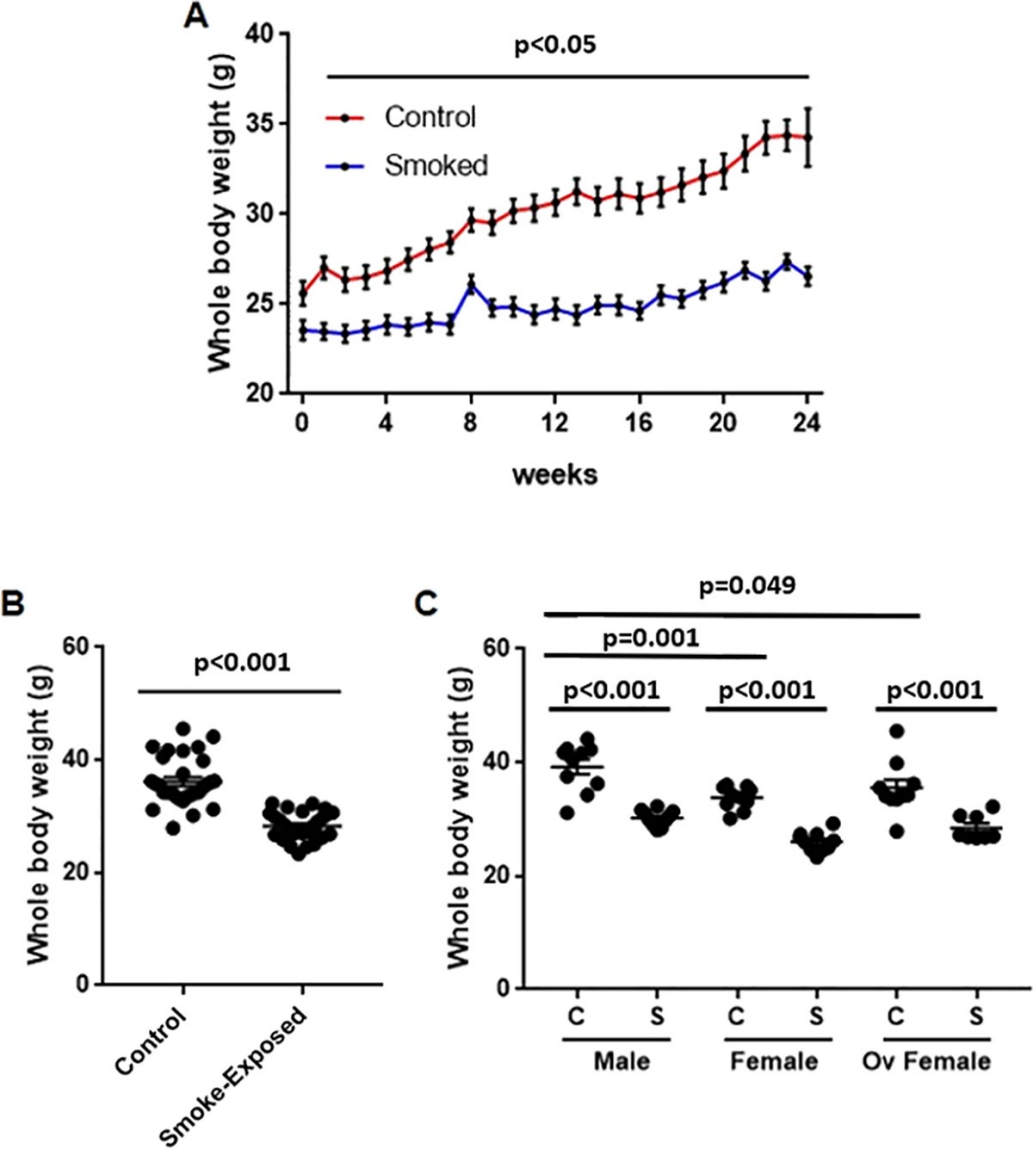
Chronic smoke exposure reduced the ability to gain body weight in mice. **A)** Whole-body weight over time is shown in mice stratified by smoke exposure (control vs. smoke-exposed). Whole-body weights at week 24 are shown in mice stratified by smoke exposure **(B)** and by both smoking and sex **(C).** In panel A, a two-way analysis of variance (ANOVA) with Bonferroni’s multiple comparisons test was applied, whereas in panels B and C a two-tailed unpaired t-test and one-way ANOVA with Bonferroni’s multiple comparisons test was used, respectively. C = control; S = smoke-exposed.

Chronic smoke exposure consistently reduced the ability to gain weight when compared to controls over the entire length of exposure (Fig 1A). Cross-sectional data at week 24 revealed that smoke exposure significantly lowered body weight compared to their control counterparts (Fig 1B), and the effect from smoking was consistent after stratification by sex in males, females and ovariectomized mice (Fig 1C). We detected significantly lower body weight in control females compared to control males. Ovariectomy did not reverse this effect, as control ovariectomized females also showed lower body weight compared to their male counterparts (Fig 1C).

### Sequencing of 16S rRNA V4 region amplicon libraries

To further characterize the contribution of the cecal microbiome on the effects of smoking on body weight, extensive bacterial sequencing technology was performed. A total of 1,217,769 reads were imported into QIIME2^®^. Following sequence quality control (DADA2), 1,017,016 reads were retained and after additional filtering steps, as outlined in the Methods section, 962,340 reads (872 different ASVs) were considered in the final analysis. Overall, the three most abundant phyla, expressed as median [interquartile range], were *Bacteroidetes* (present in 58 samples, 58.6% [11.2%]), *Firmicutes* (58 samples, 34.9% [8.8%]), and *Epsilonbacteraeota* (51 samples, 2.9% [3.2%]) (S2 Fig and S2 Table). At the genus level, the three most frequent taxa, observed across all samples, were *Prevotellaceae-UGC-001* (17.9% [11.1%]), *Lachnospiraceae NK4A136 group* (13.6% [7.4%]), and *Alistipes* (3.9% [5.0%]) (S3 Fig and S3 Table).

### Microbial community structures were different after chronic smoke exposure

**Fig 2:**
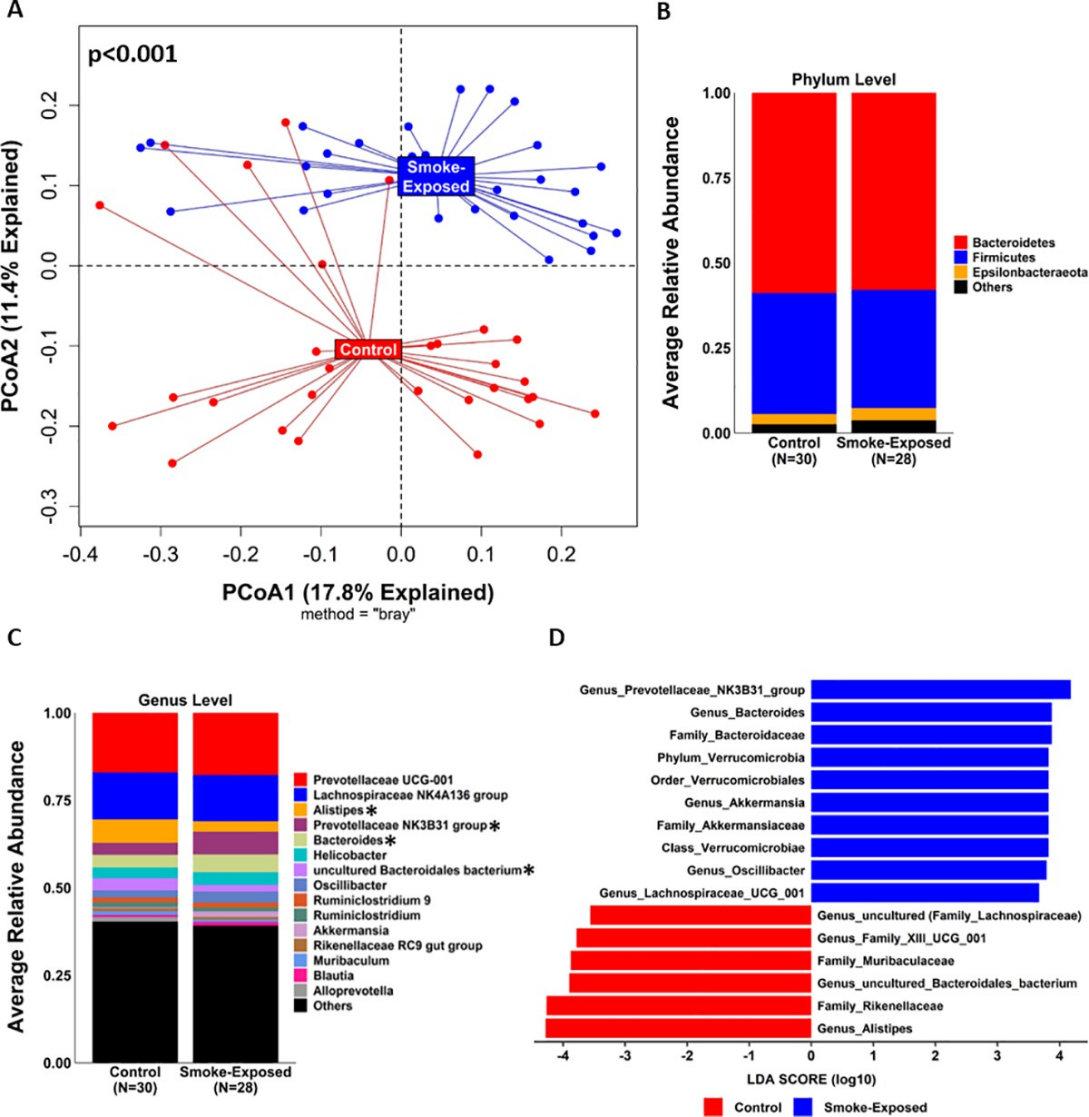
Chronic cigarette smoke exposure differentially impacted cecal microbial communities in mice. **A)** PCoA plot based on Bray-Curtis distance matrix between control (n = 30) and smoke-exposed (n = 28) samples is shown. P-value obtained using the PERMANOVA built-in function from QIIME2^®^’s diversity plugin. **B and C)** Relative abundance of most frequent taxa in control and smoke-exposed samples at the phylum and genus levels, respectively. Significant taxa differences (adj. p-value <0.05 based on the Benjamini-Hochberg method) were observed only at the genus level (*): *Alistipes* and *Uncultured Bacteroidales bacterium (Family Muribaculaceae*) showed a higher relative abundance in control samples, whereas *Prevotellaceae NK3B31group* and *Bacteroides* predominated in smoke-exposed samples. **D)** Differential taxa features identified by LEfSe (LDA score > 3.5) according to smoke exposure; red and blue bars represent taxa features with higher expression in the control and smoke-exposed groups, respectively. PCoA = Principal Component.

**Fig 3:**
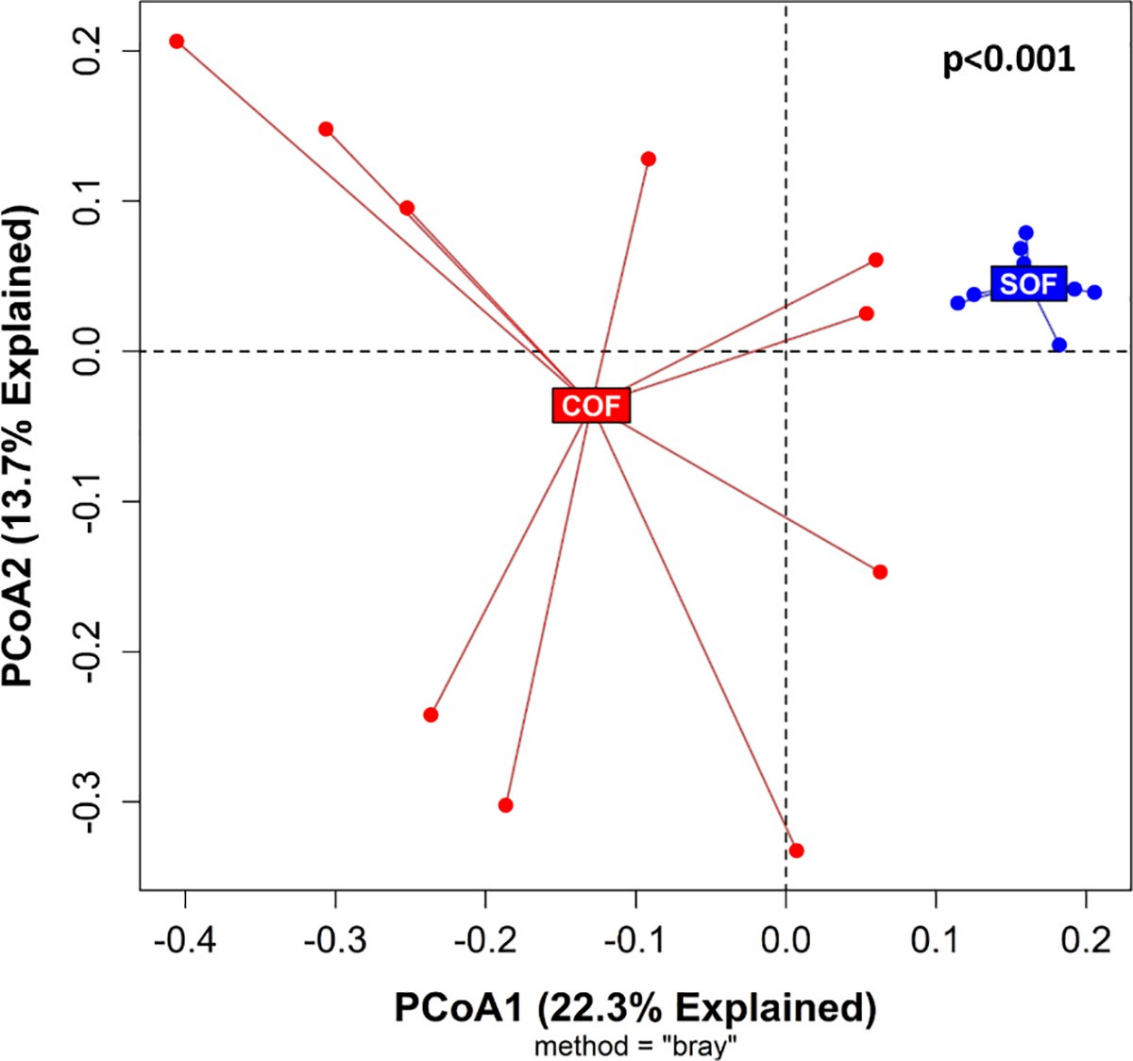
Comparison of mice cecal microbial communities between control and smoke-exposed ovariectomized females. PCoA plot based on Bray-Curtis distance matrix between control ovariectomized (COF, n = 10) and smoke-exposed ovariectomized (SOF, n = 8) female mice is shown. P-value was obtained using the PERMANOVA built-in function from QIIME2^®^’s diversity plugin. PCoA = Principal Component.

Similar values of Richness (180 [28] vs. 170 [34], p = 0.23), Shannon Index (5.8 [0.3] vs. 5.7 [0.6], p = 0.34), Evenness (0.78 [0.07] vs. 0.76 [0.06], p = 0.19), and Faith's Phylogenetic Diversity (12.4 [1.0] vs. 12.6 [0.8], p = 0.92) were observed between control (n = 30) and smoke-exposed (n = 28) samples, respectively (S4A–S4D Fig, S1 Dataset). However, the microbial community structures (beta diversity) between these groups differed significantly (Fig 2A), p<0.001. At the phylum level, relative abundance comparisons did not show significant taxa differences between control and smoke-exposed groups (Fig 2B and S4 Table). At a lower taxonomic level (genus), we observed a significantly higher RA of *Alistipes* (7.4% [4.3%] vs. 2.9% [1.5%], adj. p<0.001) and *Uncultured Bacteroidales bacterium* (Family *Muribaculaceae*) (2.4% [3.1%] vs. 1.1% [2.0%]; adj. p = 0.02) in control samples (Fig 2C, S5 Table). Conversely, the genera *Prevotellaceae NK3B31 group* (6.4% [4.5%] vs. 2.7% [4.3%], adj. p = 0.01) and *Bacteroides* (5.1% [2.9%] vs. 2.9% [3.6%], adj. p = 0.03) predominated in the smoke-exposed samples. LEfse analysis (based on an LDA score ≥ 3.5) reported similar results and identified 12 additional taxa discriminating features (total = 16) across different taxonomic levels (from phylum to genus) between both groups (Fig 2D). When considering only ovariectomized females, the effects of smoking on the cecal microbiome becomes evident, with control ovariectomized females showing significantly different microbial communities compared to their smoke-exposed counterparts (p<0.001, Fig 3).

### Female sex hormones affected cecal microbiome composition

**Fig 4:**
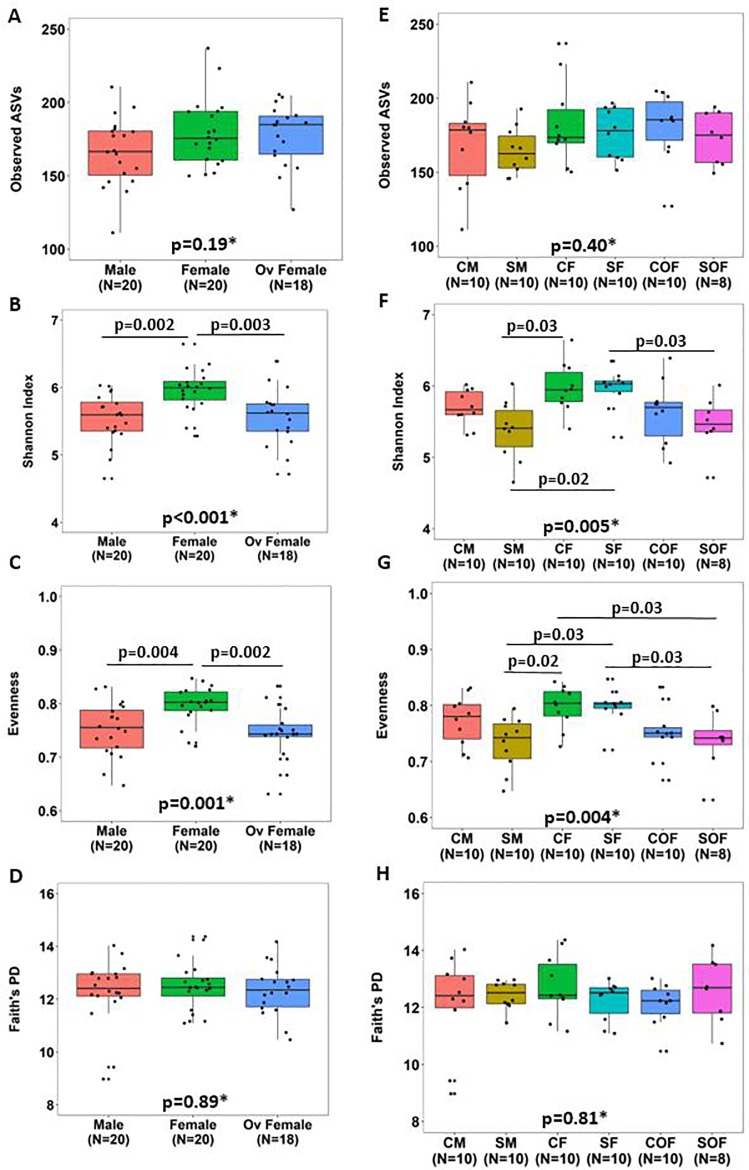
Effects of smoke exposure and sex on different alpha diversity metrics in mice cecal content. Comparisons of different alpha diversity metrics between male (n = 20), female (n = 20) and ovariectomized females (n = 18) mice (A-D) and after stratification by smoking exposure and sex (E-H) are shown. P-values were obtained using the Kruskal-Wallis test (*), and the Benjamini-Hochberg method was applied to compute adjusted p-values for multiple pairwise comparisons. ASV = amplicon sequence variant, Faith’s PD = Faith’s Phylogenetic Diversity, CM = control male, SM = smoke-exposed male, CF = control female, SF = smoke-exposed female, COF = ovariectomized control female, and SOF = ovariectomized smoke-exposed female.

Higher values of the Shannon Index were observed in female mice compared to males and ovariectomized females (6.0 [0.3] vs. 5.6 [0.4] vs. 5.6 [0.4], respectively; adj. p = 0.002 vs. males; adj. p = 0.003 vs. ovariectomized females). Similar results were also detected according to Evenness, with female mice showing significantly higher values (0.80 [0.03]) compared to males (0.76 [0.07], adj. p = 0.004) and ovariectomized females (0.74 [0.02], adj. p = 0.002) (Fig 4A–4D and S6 Table). Richness and Faith’s PD did not differ between sex groups. Interestingly, male and ovariectomized female groups showed similar values of alpha diversity for any of the metrics evaluated (adj. p >0.05).

Additional comparisons between samples after stratification by smoking and sex are shown in Fig 4E–4H and S7 Table. Values of Richness and Faith’s PD were similar across groups. We detected significantly higher values of the Shannon Index (6.0 [0.1] vs. 5.4 [0.5], adj. p = 0.02) and Evenness (0.80 [0.01] vs. 0.74 [0.06], adj. p = 0.03) in smoke-exposed female mice when compared to smoke-exposed males. Similar differences were also identified between smoke-exposed females and their ovariectomized counterparts, with the latter group showing significantly lower values of the Shannon Index (5.5 [0.3], adj. p = 0.03) and Evenness (0.74 [0.03], adj. p = 0.03). Moreover, males and ovariectomized females, regardless of smoke exposure, showed similar patterns of alpha diversity (adj. p >0.05 for all metrics).

**Fig 5:**
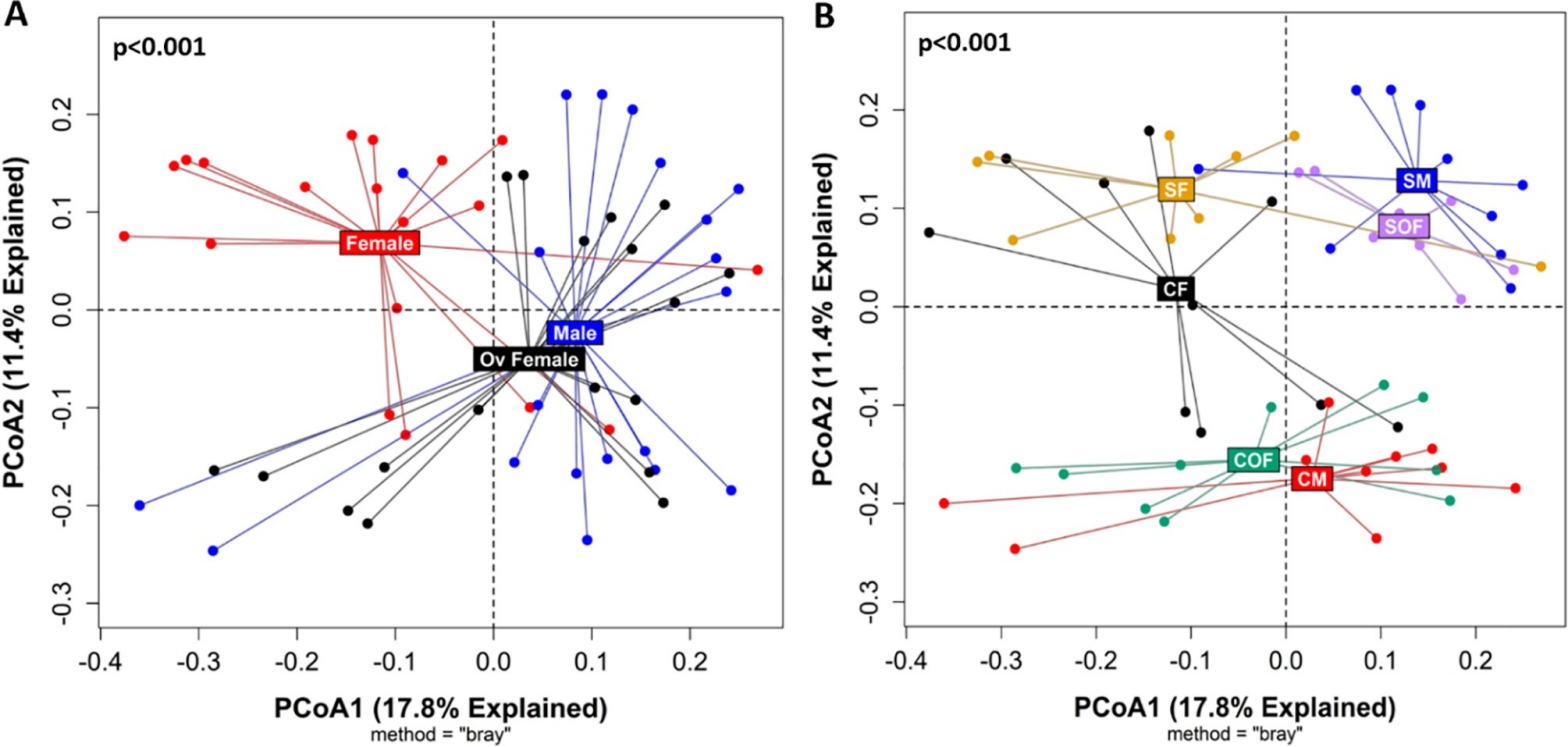
Sex hormones significantly affected the cecal microbial composition. **(A)** PCoA plot based on Bray-Curtis distance matrix between male (n = 20), female (n = 20) and ovariectomized females (Ov Female, n = 18) mice is shown. Pairwise comparisons: female vs. ovariectomized female: adj. p = 0.002; female vs. male: adj. p = 0.002; ovariectomized female vs. male: adj. p = 0.10. **(B)** PCoA plot using the same samples after stratification by smoke exposure and sex: smoke-exposed female (SF, n = 10), smoke-exposed male (SM, n = 10), ovariectomized smoke-exposed female (SOF, n = 8), control female (CF, n = 10), control male (CM, n = 10), and ovariectomized control female (COF, n = 10) is shown. Main pairwise comparisons: control female and smoked-exposed female: adj. p-value = 0.004; control male and smoked-exposed male: adj. p-value = 0.002; ovariectomized control female and ovariectomized smoke-exposed female, adj. p-value = 0.002; control female and ovariectomized control female; adj. p-value = 0.002; smoke-exposed female vs. ovariectomized smoke-exposed female: adj. p = 0.003; control male and ovariectomized control female, adj. p-value = 0.10, and smoked-exposed male and ovariectomized smoke-exposed female, adj. p-value = 0.01. A list of all multiple pairwise comparisons is described in the S8 Table. All P-values were obtained using the PERMANOVA built-in function from QIIME2^®^’s diversity plugin with adjustments for multiple pairwise comparisons according to the Benjamini-Hochberg method. PCoA = Principal Component, Ov Female = ovariectomized female.

Beta diversity analyses revealed that the cecal microbial community of female mice was significantly different from males and ovariectomized females (adj. p = 0.002 for both comparisons), with the latter two groups showing similar microbial profiles (adj. p = 0.10) (Fig 5A). When considering both smoke exposure and sex simultaneously, we identified four different microbial clusters: control females, control males/control ovariectomized females, smoke-exposed females, and smoke-exposed males/ smoke-exposed ovariectomized females (Fig 5B). These analyses revealed that the microbial community structures of control ovariectomized females were significantly different from control females (adj. p = 0.002) but similar to those of control males (adj. p = 0.10). However, among smoke-exposed samples, ovariectomized females showed significantly different microbial profiles compared to females (adj. p = 0.003) and were marginally different from males (adj. p = 0.01). S8 Table provides additional information regarding beta diversity comparisons according to smoke exposure and sex.

**Fig 6:**
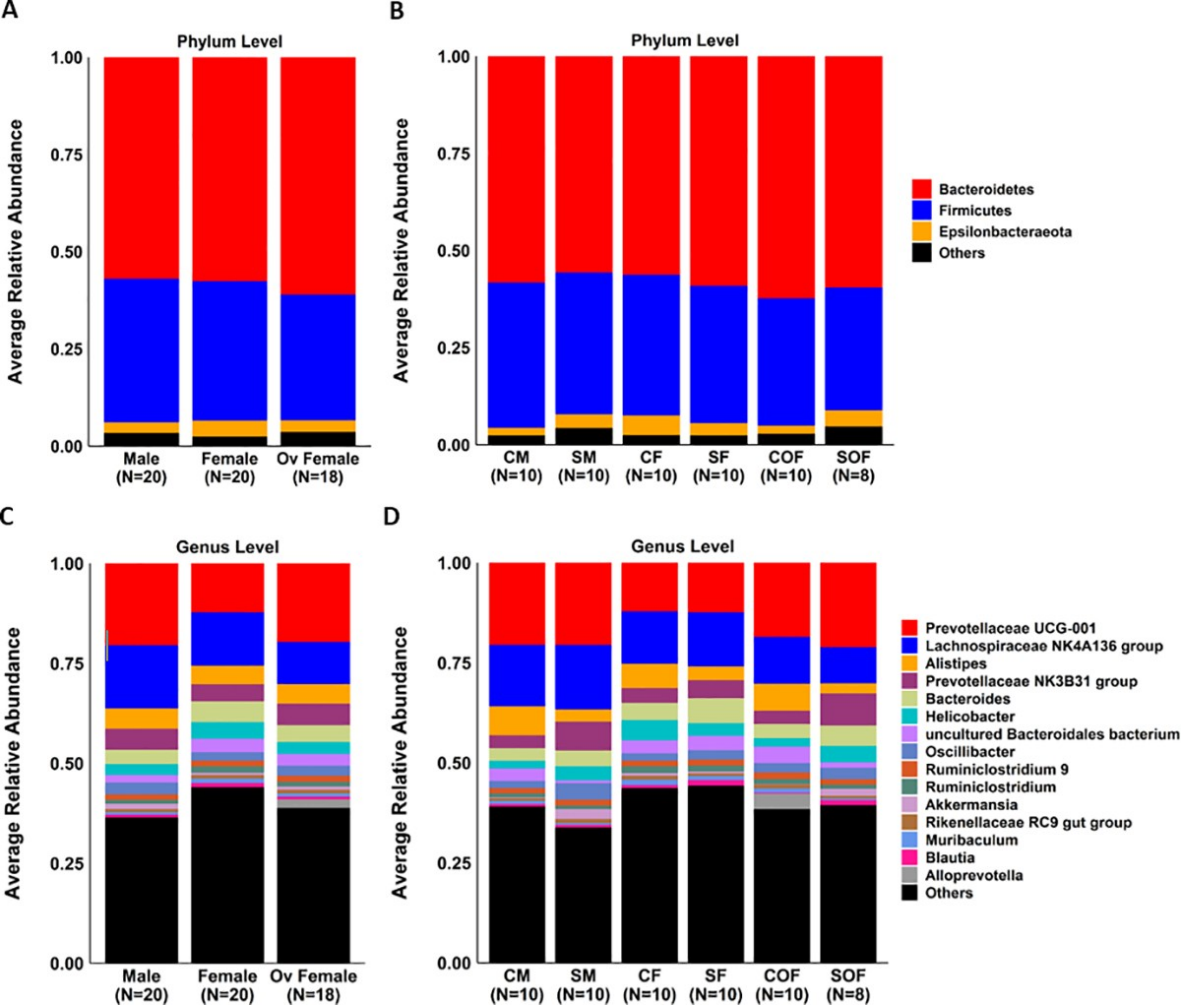
Effects of smoke exposure and sex on the phylum and genus level in mice cecal content. Average relative abundance of most frequent taxa at the phylum **(A and B)** and genus **(C and D)** levels in males, females and ovariectomized females (left) and after stratification by smoke exposure and sex (right). Ov Female = Ovariectomized Female, CM = control male, SM = smoke-exposed male, CF = control female, SF = smoke-exposed female, COF = ovariectomized control female, and SOF = ovariectomized smoke-exposed female. Taxa comparisons are described in detail in S9–S12 Tables.

**Fig 7:**
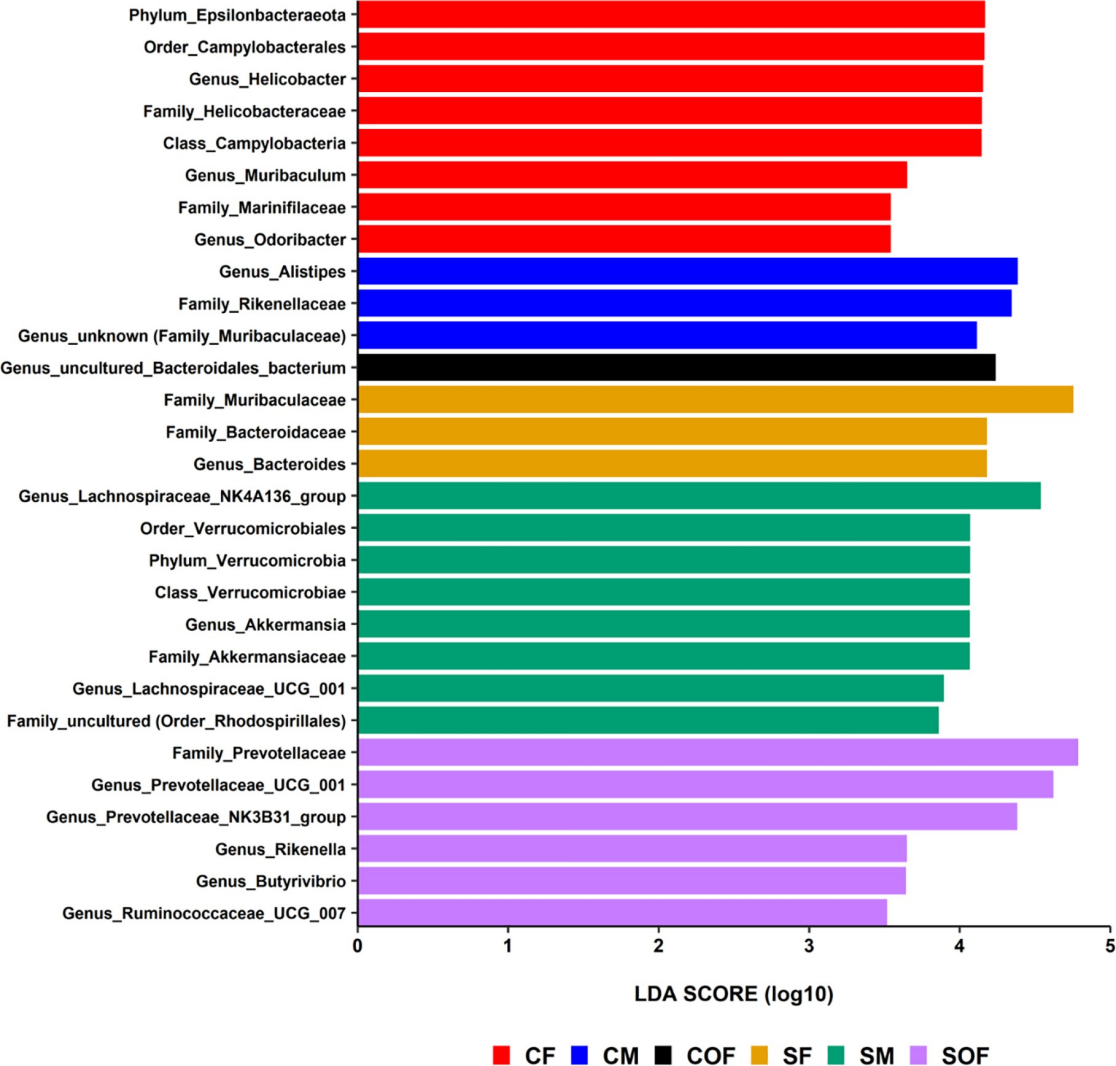
Differential taxa features identified by LEfSe after stratification by smoke exposure and sex. CF = control female, CM = control male, COF = ovariectomized control female, SF = smoke-exposed female, SM = smoke-exposed male, and SOF = ovariectomized smoke-exposed female.

These differences in microbial community structures described above were due to several taxa, especially at the genus level (Fig 6A–6D, and S9–S12 Tables). Moreover, LEfse analysis (based on an LDA score > 3.5) identified 18 differential features across groups according to sex (S5 Fig), which increased to 29 taxa differences after stratification by smoking and sex (Fig 7). According to this bioinformatics tool, the most discriminating taxa at the genus level were *Alistipes* (enriched in control males), *Lachnospiraceae NK4A136 group* and *Akkermansia* (smoke-exposed males), *Helicobacter* and *Muribaculum* (control females), *Bacteroides* (smoke-exposed females). *Prevotellaceae UCG001* and *NK3B31group* predominated in the cecal content of smoke-exposed ovariectomized females.

### Whole-body weight correlated positively with the cecal *Alistipes* expression

**Fig 8:**
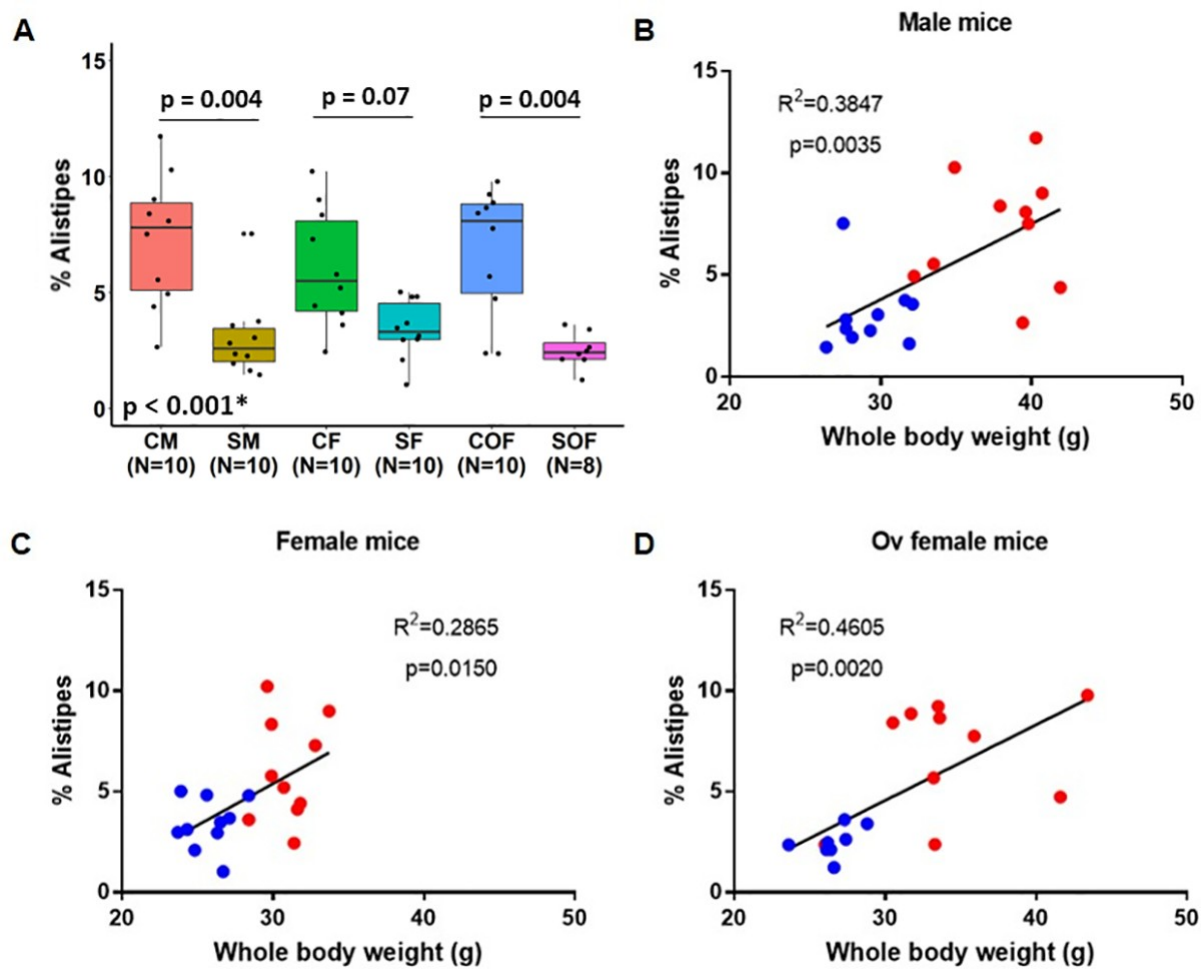
Chronic smoke-induced weight loss is associated with reduced expression of the *Alistipes* genus in the cecal content. **A)** Relative abundance of the bacterial genus *Alistipes* in mice stratified by smoke exposure and sex at 24 weeks post cigarette smoke exposure; p-value* was obtained using the Kruskal-Wallis test, and the Benjamini-Hochberg method was applied to compute adjusted p-values for multiple pairwise comparisons. Correlations between whole-body weight and the cecal relative abundance of *Alistipes* (%) are shown in male **(B)**, female **(C),** and ovariectomized female **(D)** mice. Linear regression analyses were used in panels B-D. Red dot = control mice, blue dot = smoke-exposed mice. C = control, S = smoke-exposed, Ov Female = ovariectomized female.

We detected a significant decrease in the *Alistipes* expression in the cecal content of male and ovariectomized female mice after chronic smoking exposure (adj. p = 0.004 for both comparisons). Among females, a similar trend was also observed (adj. p = 0.07). Interestingly, this effect was positively correlated with body weight in all sex groups (males, females, and ovariectomized females) (Fig 8A–8D, S2 Dataset). Among the ASVs belonging to the genus *Alistipes* (n = 16), predicted hits at the species level according to the NCBI sequence BLAST tool [41] included *A*. *finegoldii* (E-value: 2e^-107^; Identity: 100%) and *A*. *obesi* strain ph8 16S ribosomal RNA (E-value: 9e^-105^, Identity: 99.1%).

## Discussion

We have previously demonstrated that female mice have increased airway wall thickness, lymphoid follicle expression, oxidative stress and distal airway resistance after chronic smoke exposure compared to male mice, and these effects were attenuated with ovariectomy [23, 24, 26]. To extend previous findings that showed chronic cigarette smoke exposure altered the murine gut microbiome [42], we have systemically investigated specific sex-related effects in the cecal microbiome of mice in response to chronic smoke exposure. According to our data, chronic smoke exposure significantly altered the cecal microbial compositions of male, female and ovariectomized mice compared to their respective controls. However, the most novel finding was that ovariectomy can significantly reverse the effect of female sex hormones on the cecal microbiome as both male and ovariectomized female mice showed similar patterns according to both alpha (Fig 4A–4D, S6 Table) and beta diversity analyses (Fig 5A). Moreover, these effects may occur regardless of whether smoke exposure was present, as the microbial communities of control males and control ovariectomized females were similar (Fig 5B), and their smoke-exposed counterparts were marginally different from each other. Collectively, these data support a significant role of sex hormones on the cecal microbial community, which may have important implications to diseases that have a sex-dependent component.

Fecal and cecal samples from murine models are often used to study the interaction between the gut microbiome and the host response. In human studies, the fecal microbiome is mostly investigated because sample collection is relatively easy, as no invasive procedures are required for sample collection. However, the cecum is an important fermentation chamber that harbors a rich diversity of microbes [43–45], which may reflect the stability observed in the microbial environment. In a previous article, Org et al. performed 16S bacterial sequencing on the cecum of 689 mice from 89 different strains [20]. These authors observed a higher abundance of *Allobaculum* and *Anaeroplasma* in the gut microbiome of males, whereas *Lachnospiraceae* (*Dorea*, *Coprococcus* and *Ruminococcus*) predominated in those of females. However, most of those differences were due to different genetic backgrounds (i.e., different strains), possibly hampering the assessment of the true biological effects of sex hormones in the gut microbiome. For instance, it has been shown that the microbiome composition of healthy mice may be affected by several factors, such as cage, shipment and vendors [46]. Interestingly, the same authors above performed gonadectomy in a subset of male mice, which led to significant changes in the gut microbiome, and those effects were mitigated with testosterone administration. Here, using controlled experimental conditions (same mice strain and diet), we provide additional and more robust evidence of the impact of female sex hormones on the cecal microbiome.

The mechanism by which sex hormones modulates the cecal microbiome is not fully understood. It has been shown that female sex hormones upregulate the expression of branched-chain 2-oxoacid dehydrogenase [47]. Shastri et al. found evidence of sex differences in gut fermentation, as female mice, after being fed on an oligofructose supplemented chow diet, evolved with increased expression of the *Bacteroidetes* phylum compared to males [19]. Interestingly, the authors also observed significantly higher weight gain and energy consumption in males compared to females regardless of the diet used (either chow or chow supplemented with oligofructose). Similarly, in our study, we observed significantly higher body weight in control male mice compared to their female counterparts. This suggests that body weight may be imprinted and partially controlled by a unique set of microbes that differ in their ability to extract and deposit nutrients in the body, however, this requires further studies to validate this hypothesis. Additionally, the exact biological role associated with each bacterial strain in the mice gut remains unclear, however, increasing evidence supports that the entire gut microbiome in conventional mice compared to germ-free has a global overexpression of metabolites that have been reported to be associated with mucosal integrity [48], energy extraction [49] and chemical detoxification [50]. Recently, Baars and colleagues observed a crosstalk between sex-induced changes in the gut microbiome and the regulation of host lipid metabolism [51]. These sex differences in energy metabolism have also been described in humans [52], but require further studies to elucidate their exact mechanisms.

Consistent with other reports [53], we also showed that chronic smoke exposure significantly reduced the ability to gain weight in smoke-exposed mice compared to control mice. Apart from the neurophysiological effects of smoking, which is associated with decreased appetite and changes in plasma leptin levels [53], smoking also interferes negatively with the gut homeostasis by promoting increased mucosal inflammation and decreased intestinal mucosal barrier integrity. In a previous study, mice exposed to two months of smoke exposure followed by a 6-day treatment with dextran sodium sulfate (DSS) (induced-colitis model) showed enhanced gut inflammation via increased neutrophil counts and Th17+ cells in the lungs, blood and intestinal compartments, supporting the existence of a lung-gut axis [54]. Those findings are in keeping with reports from Allais and colleagues, who observed altered mucin production and increased expression of inflammatory genes (Cxcl2 and IL-6) in the murine gut following chronic smoke exposure, and those changes were accompanied by an increase in the relative abundance of *Lachnospiraceae sp*. in the colon [42]. In another study that analyzed fecal samples of 758 men, a decrease in *Firmicutes/Bacteroidetes* ratio in current smokers compared to never smokers was observed [55]. We extend these observations by reporting several taxa differences at the genus level between control and smoke-exposed mice samples. We observed that the genera *Bacteroides*, *Prevotellaceae NK3B31group*, *Akkermansia*, *Oscillibacter* and *Lachnospiraceae UCG-001* predominated in the gut microbiome of smoke-exposed mice, whereas *Alistipes* and *Uncultured Bacteroidales bacterium* (Family *Muribaculaceae*) were enriched in control mice.

The shifts in the expression of the *Alistipes* genus related to chronic smoke exposure detected in our study deserve special considerations. The relative abundance of *Alistipes* was consistently decreased in all smoke-exposed mice groups compared to their respective controls, and its relative abundance was significantly and positively correlated with whole-body mice weight. Nevertheless, owing to our study design, it remains unclear whether a decreased expression of this particular genus is causally related to weight loss or merely reflects a perturbed cecal mucosal environment due to different insults from smoking. Dziarski and colleagues also detected a reduction in *Alistipes finegoldii* (identified among the *Alistipes* species in our study) in the gut microbiome of mice that harboured DSS-induced colitis [56]. Interestingly, oral delivery of *A*. *finegoldii* has been shown to protect these mice with DDS-induced colitis from excess body weight loss, as well as from adverse histologic changes in the gut including epithelial hyperplasia, loss of crypts, immune cell infiltration, ulceration, epithelial and goblet cell loss [56]. Decreased gut expression of several species belonging to the same *Alistipes* genus including *A*. *massiliensis*, *A*. *putredinis* and *A*. *finegoldii* has also been linked to inflammatory bowel disease (IBD) [57–59]. These data support that the delivery of specific bacterial strains may have therapeutic effects that are able to reverse or attenuate the inflammatory process associated with IBD. This has clinical implications, as it has been shown that smokers have an increased risk for IBD [60], while another study has reported that female smokers are at increased risk of Crohn’s disease compared to men [61]. Taken together, smoking, aside from its deleterious effects on the lungs, may also induce significant perturbations on the gastrointestinal tract. Whether these gut microbiome changes induced by smoking are causally related to different diseases, as in IBD, or represent possible disease biomarkers (e.g., decreased *Alistipes* expression) requires further studies. Similarly, it remains unknown how these smoking-induced gut microbiome shifts may reflect on lungs, however, a growing body of evidence supports the existence of a gut lung-axis [62], as gut metabolites, after reaching the lungs through the blood-stream, can induce immune and inflammatory responses. Thus, our findings may be beneficial for future studies in search of novel therapies for smoking-related diseases.

Our study has several limitations. First, although we demonstrated a positive correlation between *Alistipes* and body weight in cigarette-exposed mice, we did not establish causality. Second, the gut microbiome of mice is quantitatively different from that of humans, especially when it pertains to the relative abundance of specific phyla and species, though they do share similar qualitative cores [63]. Although the *Firmicutes* and the *Bacteroidetes* are the two abundant phyla in both humans and mice, 85% of the sequences in the gut microbiome of mice represent genera that have not been previously detected in humans [46, 64]. Thirdly, we cannot completely exclude any cage effects on our cecal microbiome analyses, as mice are generally housed in groups of 4–5 per cage. In a previous study, Ericsson and colleagues reported that different types of bedding (aspen vs paper chips) and cage ventilation conditions (static vs ventilated micro-isolator) may have effects on the gut microbiota [65], which, in turn, may interfere with the reproducibility of animal models. However, these potential cage effects were minimized in our study, as we consistently used the same bedding, diet and housing room in all our experiments. In keeping with this, we also performed additional beta diversity analyses (S6 Fig) that showed only minimal cage effects in ovariectomized females.

In summary, our data indicate that chronic smoke exposure alters the microbial community in the cecal content of mice and that these changes are also modulated by female sex hormones. Another important finding was that the relative abundance of *Alistipes* was positively associated with whole-body weight. Reduced body weight is an important risk factor for chronic lung diseases including chronic obstructive pulmonary disease and bronchiectasis. Further studies are needed to reveal the specific mechanisms, such as metabolic factors, systemic inflammatory status or hormonal milieu that drive weight loss in these conditions and to what extent these mechanisms are modulated by female sex hormones.

## Supporting information

S1 Fig Comparison of total bacterial 16S load between all cecal samples (n = 58) and extraction negative controls (n = 6). 16S DNA copies (number of copies/μL) were normalized to total DNA content in microliter (μl).
(TIF)

S2 Fig Average relative abundance of most abundant phyla across cecal samples (n = 58). “Others” category (in black) included five different phyla (*Proteobacteria*, *Cyanobacteria*, *Deferribacteres*, *Verrucomicrobia*, and *Tenericutes*), whose mean abundance was lower than 2.0% across all cecal samples.
(TIF)

S3 Fig Average relative abundance of most abundant genera across cecal samples (n = 58). “Others” category (in black) included 92 additional genera.
(TIF)

S4 Fig Comparisons of different alpha diversity metrics between control (n = 30) and smoke-exposed (n = 28) cecal samples. (Panel A–Richness; Panel B–Shannon Index, Panel C—Pielou's Evenness Index, and Panel D—Faith's Phylogenetic Diversity).
(TIF)

S5 Fig Differential taxa features identified by LEfSe according to sex (LDA score > 3.5). (TIF)

S6 Fig Effects of cages on mice cecal microbial communities. (TIF)

S1 Table Taxonomic annotations at the phylum and genus levels for potential contaminants removed from cecal samples for downstream analysis (criteria: Amplicon sequence variants present in at least two out of the six extraction negative controls and whose average relative abundance were higher compared to cecal samples). (DOCX)

S2 Table Relative abundance of most abundant phyla observed across cecal samples (n = 58). (DOCX)

S3 Table Relative abundance of most abundant genera (top 15) observed across cecal samples (n = 58). (DOCX)

S4 Table Relative taxa abundance comparisons at the phylum level between control and smoke-exposed samples. (DOCX)

S5 Table Relative taxa abundance comparisons at the genus level between control and smoke-exposed samples. (DOCX)

S6 Table Comparisons of different alpha diversity metrics between male, female and ovariectomized female groups. (DOCX)

S7 Table Comparisons of different alpha diversity metrics after stratification by smoke exposure and sex. (DOCX)

S8 Table Multiple pairwise comparisons related to beta diversity analyses after stratification by smoke exposure and sex. (DOCX)

S9 Table Relative taxa abundance comparisons at the phylum level between male, female and ovariectomized female groups. (DOCX)

S10 Table Relative taxa abundance comparisons at the phylum level after stratification by smoke exposure and sex. (DOCX)

S11 Table Relative taxa abundance comparisons at the genus level between male, female and ovariectomized female groups. (DOCX)

S12 Table Relative taxa abundance comparisons at the genus level after stratification by smoke exposure and sex. (DOCX)

S1 Dataset Values of alpha diversity metrics (Richness, Shannon Index, Evenness, and Faith’s PD) in all cecal samples (n = 58). (XLSX)

S2 Dataset Relative expression abundances (%) of the *Alistipes* genus in all cecal samples (n = 58). (XLSX)
