# Supplementary figures and images for: Effects of sex and chronic cigarette smoke exposure on the mouse cecal microbiome
Source: PLoS One. 2020 Apr 6;15(4):e0230932. doi: 10.1371/journal.pone.0230932 (PMC7135149; doi:10.1371/journal.pone.0230932)

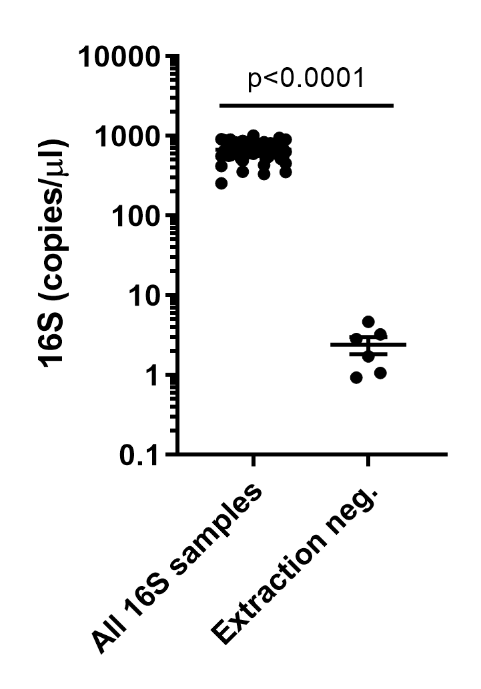

Supplement: S1 Fig — 16S DNA copies (number of copies/μL) were normalized to total DNA content in microliter (μl). (TIF) [file pone.0230932.s001.tif]

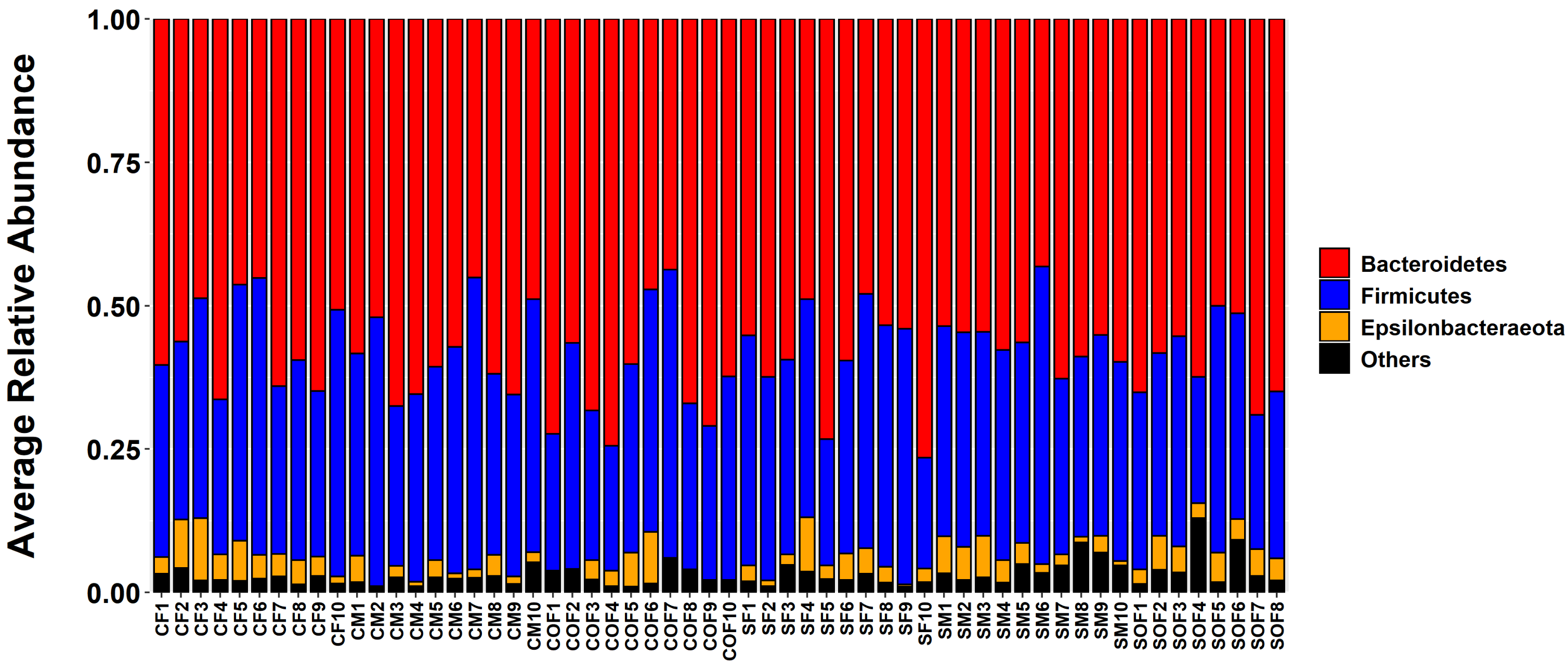

Supplement: S2 Fig — “Others” category (in black) included five different phyla (Proteobacteria, Cyanobacteria, Deferribacteres, Verrucomicrobia, and Tenericutes), whose mean abundance was lower than 2.0% across all cecal samples. (TIF) [file pone.0230932.s002.tif]

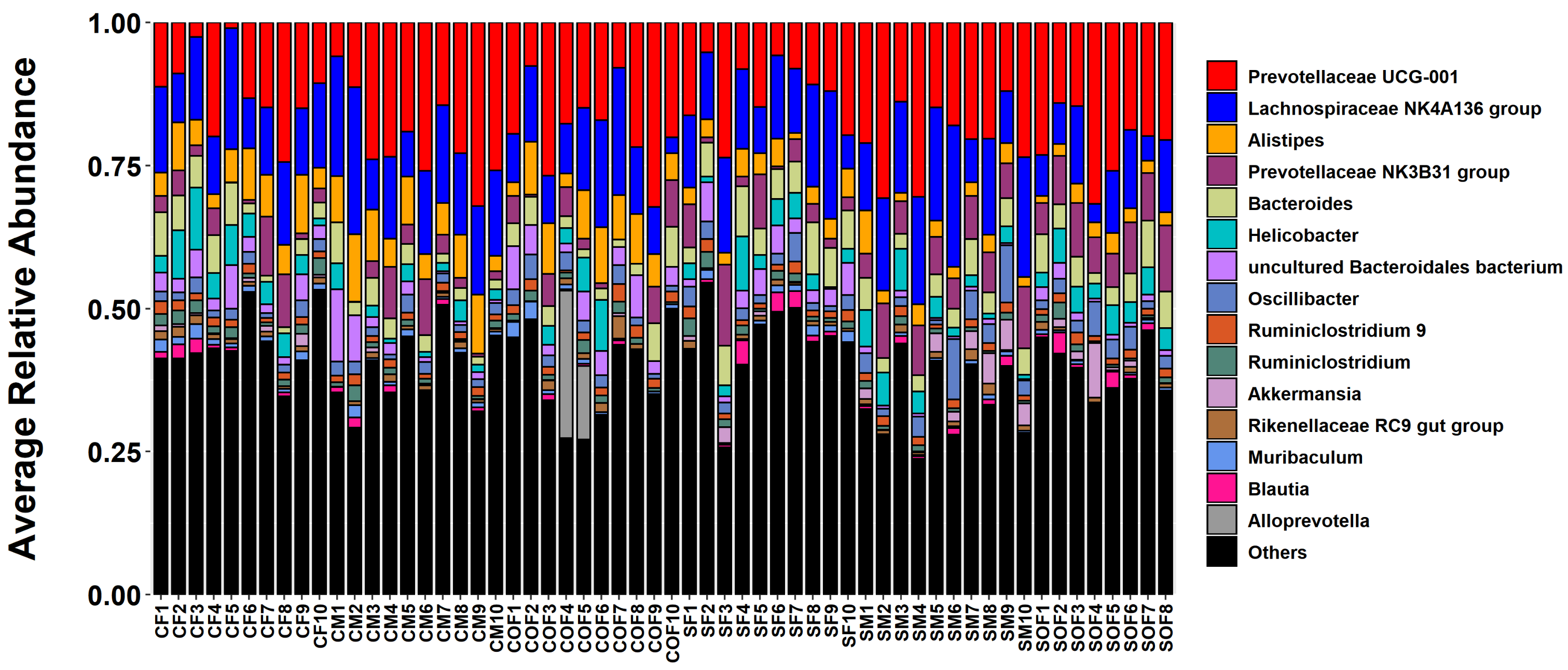

Supplement: S3 Fig — “Others” category (in black) included 92 additional genera. (TIF) [file pone.0230932.s003.tif]

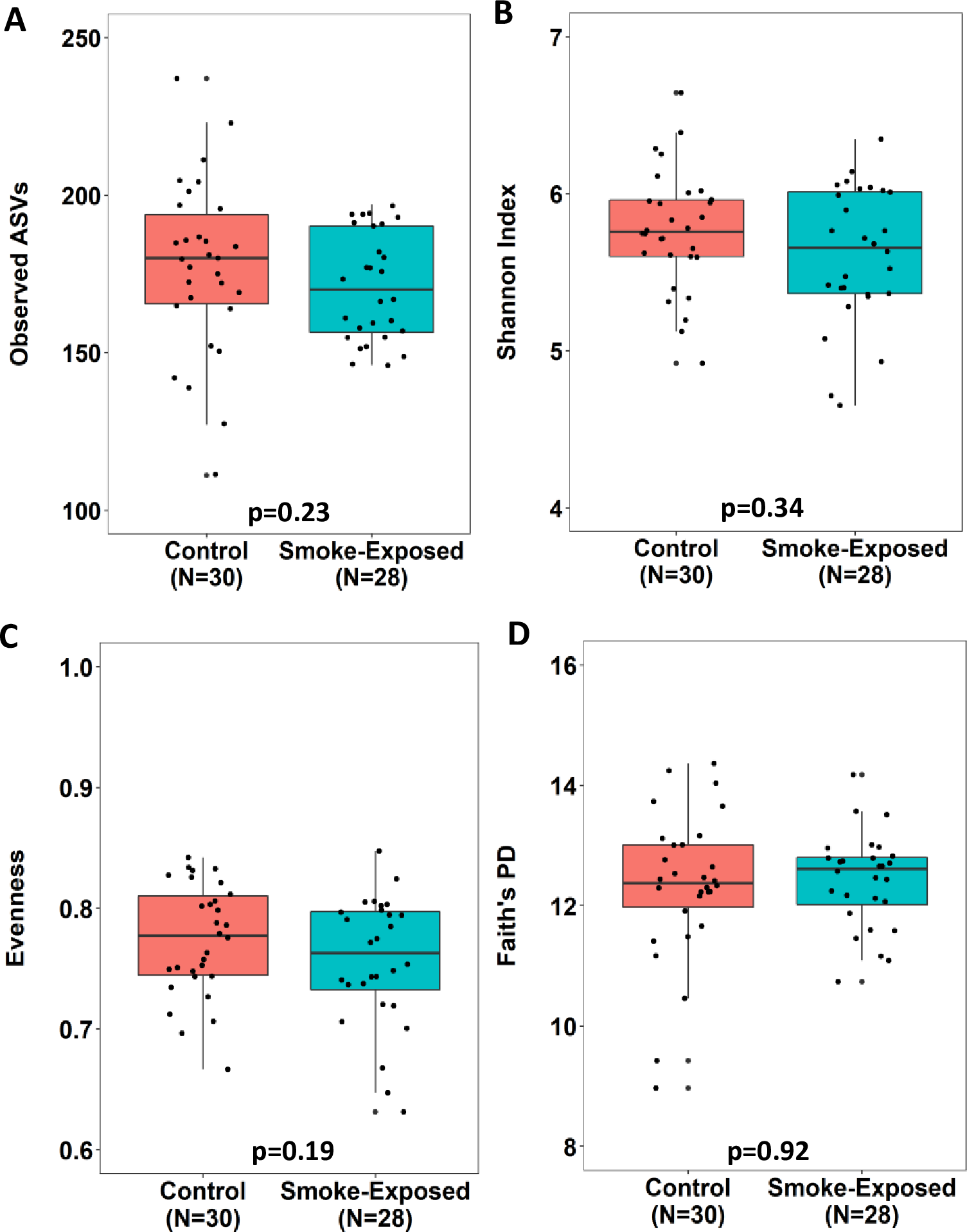

Supplement: S4 Fig — (Panel A–Richness; Panel B–Shannon Index, Panel C—Pielou's Evenness Index, and Panel D—Faith's Phylogenetic Diversity). (TIF) [file pone.0230932.s004.tif]

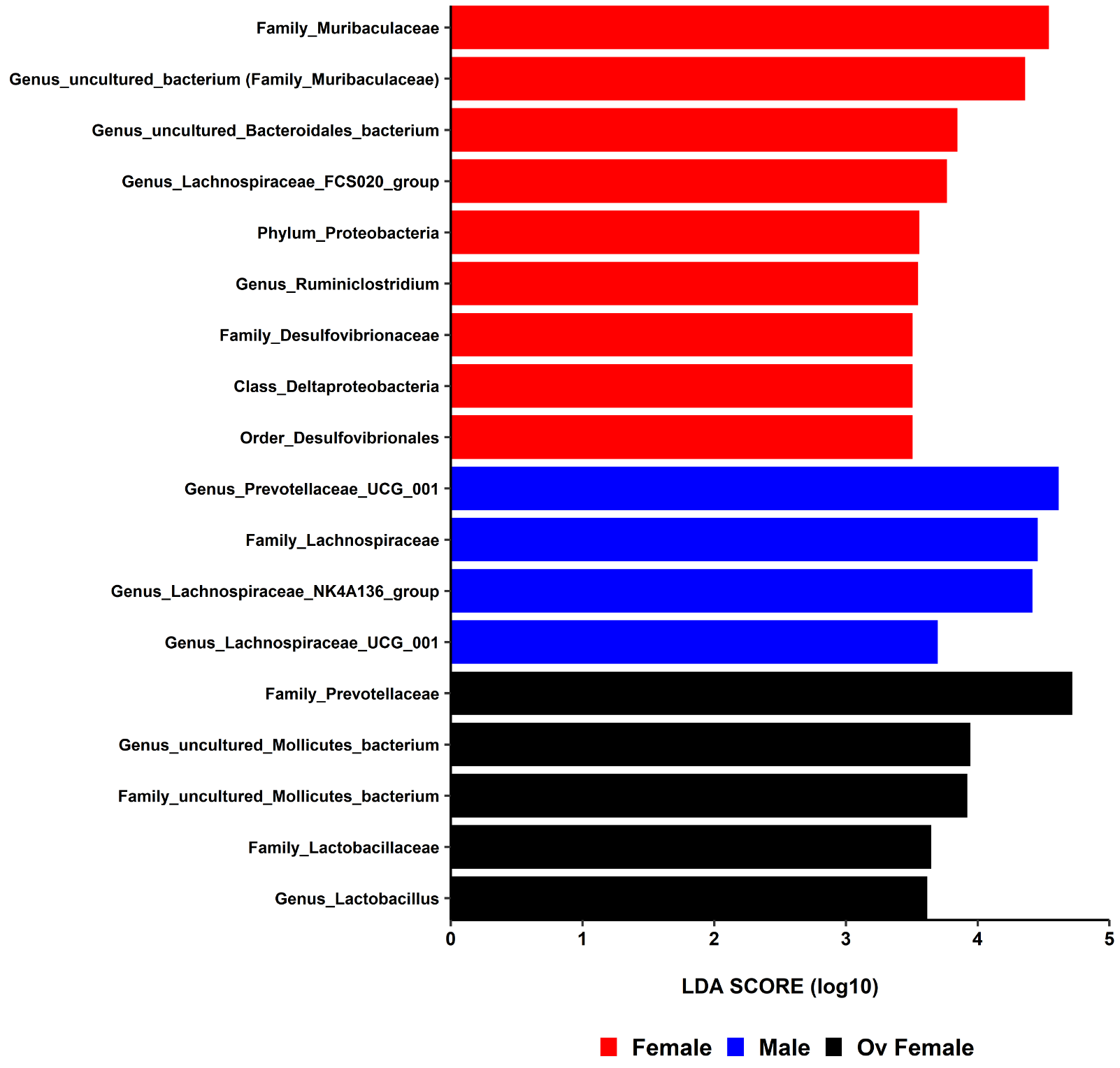

Supplement: S5 Fig — (TIF) [file pone.0230932.s005.tif]

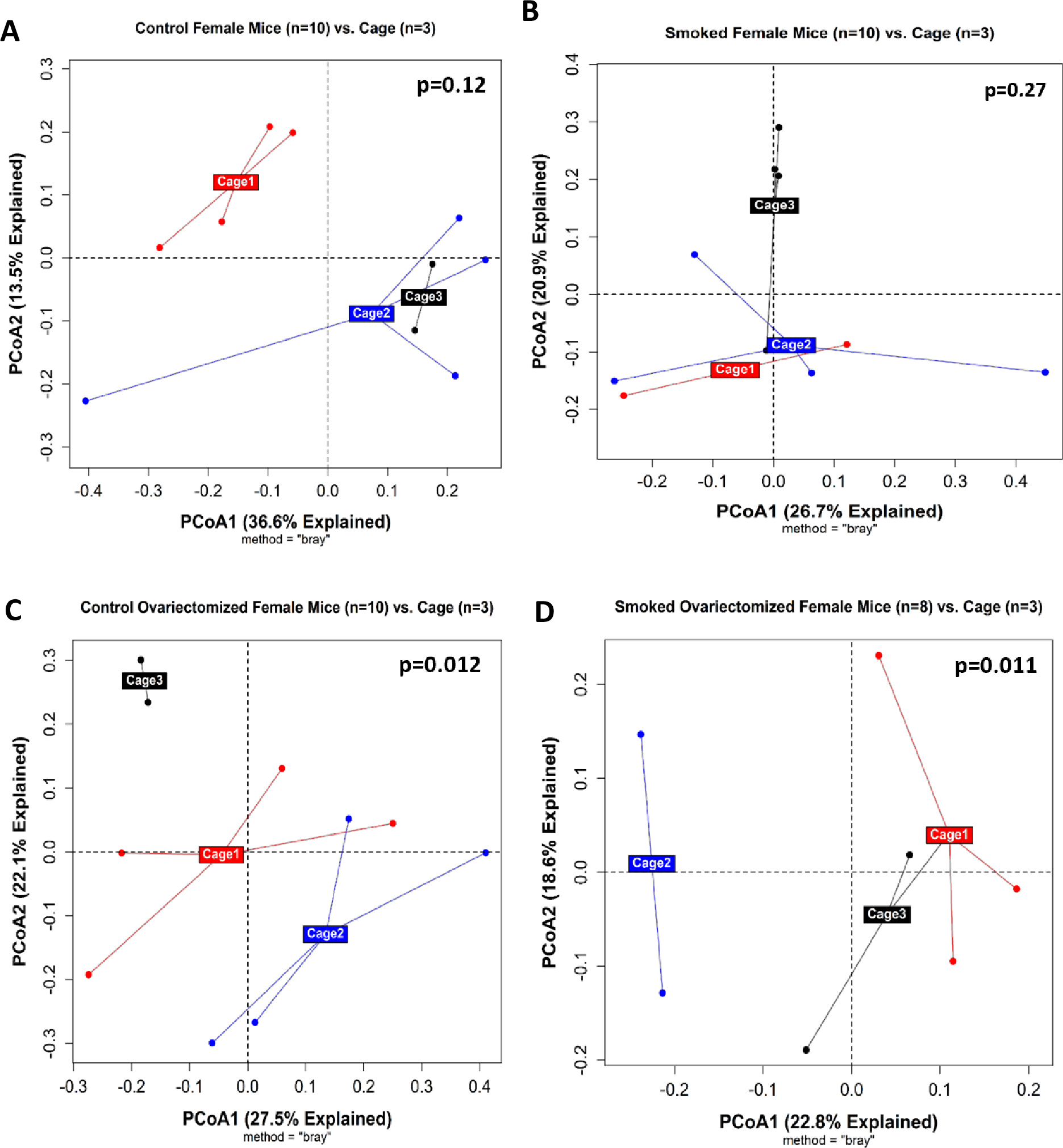

Supplement: S6 Fig — (TIF) [file pone.0230932.s006.tif]
