# Supplementary material for: Effects of sex and chronic cigarette smoke exposure on the mouse cecal microbiome
Source: PLoS One. 2020 Apr 6;15(4):e0230932. doi: 10.1371/journal.pone.0230932 (PMC7135149; doi:10.1371/journal.pone.0230932)
Supplement: S1 Table — (DOCX) [file pone.0230932.s007.docx]

**S1 Table.** **Taxonomic annotations at the phylum and genus levels for potential contaminants removed from cecal samples** **for downstream analysis (criteria: amplicon sequence variants present in at least two out of the six extraction negative controls and whose average relative abundance were higher compared to cecal samples).**

| **Number of times**  **observed** | **# of Controls**  **observed in** | **Phylum*** | **Genus*** |
| --- | --- | --- | --- |
| 4,745 | 6 | *Bacteroidetes* | *Flavobacterium* |
| 3,474 | 3 | *Proteobacteria* | Not assigned |
| 3,251 | 3 | *Bacteroidetes* | *Cloacibacterium* |
| 1,042 | 2 | *Firmicutes* | *uncultured* |
| 974 | 4 | *Bacteroidetes* | *uncultured Bacteroidales bacterium* |
| 577 | 3 | *Firmicutes* | *Lachnospiraceae NK4A136 group* |
| 525 | 4 | *Firmicutes* | *Lactobacillus* |
| 509 | 3 | *Bacteroidetes* | Not assigned |
| 448 | 2 | *Cyanobacteria* | Not assigned |
| 409 | 2 | *Proteobacteria* | *Acinetobacter* |
| 369 | 2 | *Bacteroidetes* | *Odoribacter* |
| 329 | 2 | *Bacteroidetes* | *uncultured bacterium* |
| 286 | 2 | *Bacteroidetes* | *uncultured bacterium* |
| 273 | 2 | *Bacteroidetes* | *uncultured bacterium* |
| 253 | 3 | *Proteobacteria* | *Pseudomonas* |
| 247 | 3 | *Proteobacteria* | *Pseudomonas* |
| 228 | 2 | *Bacteroidetes* | *uncultured Bacteroidales bacterium* |
| 218 | 2 | *Bacteroidetes* | *Ambiguous_taxa* |
| 72 | 2 | *Actinobacteria* | *Not assigned* |
| 34 | 3 | *Proteobacteria* | *Escherichia-Shigella* |
| 24 | 3 | *Actinobacteria* | *Bifidobacterium* |

*According to the Silva rRNA database (v132)
