# Supplementary material for: Effects of sex and chronic cigarette smoke exposure on the mouse cecal microbiome
Source: PLoS One. 2020 Apr 6;15(4):e0230932. doi: 10.1371/journal.pone.0230932 (PMC7135149; doi:10.1371/journal.pone.0230932)
Supplement: S2 Table — (DOCX) [file pone.0230932.s008.docx]

**S2 Table. Relative abundance of most abundant phyla observed across cecal samples (n=58).**

| **Genus** | **Relative Abundance** | | |
| --- | --- | --- | --- |
|  | **Median** | **IQR** | **Range** |
| ***Bacteroidetes, %*** | **58.6** | **11.2** | **43.2 – 76.5** |
| ***Firmicutes*, %** | **34.9** | **8.8** | **19.3 – 51.9** |
| ***Epsilonbacteraeota*, %** | **2.9** | **3.2** | **0 – 10.9** |

Legend: IQR: interquartile range
