# Supplementary material for: Effects of sex and chronic cigarette smoke exposure on the mouse cecal microbiome
Source: PLoS One. 2020 Apr 6;15(4):e0230932. doi: 10.1371/journal.pone.0230932 (PMC7135149; doi:10.1371/journal.pone.0230932)
Supplement: S3 Table — (DOCX) [file pone.0230932.s009.docx]

**S3 Table.** **Relative abundance of most abundant genera (top 15) observed across cecal samples (n=58).**

| **Genus** | **Relative Abundance** | | |
| --- | --- | --- | --- |
|  | **Median** | **IQR** | **Range** |
| ***Prevotellaceae UCG001*, %** | **17.9** | **11.1** | **0.9 – 32.3** |
| ***Lachnospiraceae NK4A136 group*, %** | **13.6** | **7.4** | **2.8 – 25.7** |
| ***Alistipes*, %** | **3.9** | **5.0** | **1.0 – 11.7** |
| ***Prevotellaceae NK3B31group*, %** | **4.7** | **6.2** | **0.0 – 14.2** |
| ***Bacteroides*, %** | **4.1** | **3.6** | **1.0 – 9.1** |
| ***Helicobacter*, %** | **2.9** | **3.2** | **0.0 – 10.9** |
| ***Uncultured Bacteroidales bacterium*, %** | **2.0** | **2.4** | **0.3 – 12.7** |
| ***Oscillibacter*, %** | **2.1** | **1.7** | **0.8 – 10.6** |
| ***Ruminiclostridium 9, %*** | **1.4** | **0.5** | **0.4 – 3.1** |
| ***Ruminiclostridium*, %** | **1.1** | **0.8** | **0.2 – 3.1** |
| ***Akkermansia*, %** | **0.1** | **1.0** | **0.0 – 9.6** |
| ***Rikenellaceae RC9 gut group*, %** | **0.8** | **0.6** | **0.0 – 3.9** |
| ***Muribaculum*, %** | **0.7** | **0.7** | **0.0 – 3.1** |
| ***Blautia*, %** | **0.5** | **0.9** | **0.0 – 4.2** |
| ***Alloprevotella*, %** | **0** | **0** | **0 – 25.8** |

Legend: IQR: interquartile range
