# Supplementary material for: Effects of sex and chronic cigarette smoke exposure on the mouse cecal microbiome
Source: PLoS One. 2020 Apr 6;15(4):e0230932. doi: 10.1371/journal.pone.0230932 (PMC7135149; doi:10.1371/journal.pone.0230932)
Supplement: S4 Table — (DOCX) [file pone.0230932.s010.docx]

**S4 Table.** **Relative taxa abundance comparisons at the phylum level between control and smoke-exposed samples.**

| **Phylum** | **Control samples**  **(n=30)** | **Smoke-exposed samples**  **(n=28)** | **P-value*** | **Adjusted**  **P-value**^†^ |
| --- | --- | --- | --- | --- |
| ***Bacteroidetes*, %** | **60.3 [14.5]** | **57.1 [8.6]** | **0.46** | **0.69** |
| ***Firmicutes*, %** | **33.6 [12.6]** | **35.2 [6.3]** | **0.89** | **0.89** |
| ***Epsilonbacteraeota*, %** | **2.8 [3.4]** | **3.3 [2.4]** | **0.18** | **0.53** |

Values expressed as median [interquartile range]. *P-values obtained using the Wilcoxon rank-sum test. ^†^Adjusted P-values were determined using the Benjamini-Hochberg method.
