# Supplementary material for: Effects of sex and chronic cigarette smoke exposure on the mouse cecal microbiome
Source: PLoS One. 2020 Apr 6;15(4):e0230932. doi: 10.1371/journal.pone.0230932 (PMC7135149; doi:10.1371/journal.pone.0230932)
Supplement: S5 Table — (DOCX) [file pone.0230932.s011.docx]

**S5 Table.** **Relative taxa abundance comparisons at the genus level between control and smoke-exposed samples.**

| **Genus** | **Control samples**  **(n=30)** | **Smoke-exposed samples**  **(n=28)** | **P-value*** | **Adjusted**  **P-value**^†^ |
| --- | --- | --- | --- | --- |
| ***Prevotellaceae UCG001*, %** | **137.4 [12.1]** | **18.4 [8.2]** | **0.83** | **0.83** |
| ***Lachnospiraceae NK4A136 group*, %** | **14.3 [6.2]** | **13.1 [7.8]** | **0.83** | **0.83** |
| ***Alistipes*, %** | **7.4 [4.3]** | **2.9 [1.5]** | **<0.001** | **<0.001** |
| ***Prevotellaceae NK3B31 group*, %** | **2.7 [4.3]** | **6.4 [4.5]** | **0.001** | **0.01** |
| ***Bacteroides*, %** | **2.9 [3.6]** | **5.1 [2.9]** | **0.008** | **0.03** |
| ***Helicobacter*, %** | **2.8 [3.4]** | **3.3 [2.4]** | **0.18** | **0.26** |
| ***Uncultured Bacteroidales bacterium*, %** | **2.4 [3.1]** | **1.1 [2.0]** | **0.004** | **0.02** |
| ***Oscillibacter*, %** | **2.0 [1.0]** | **2.6 [1.9]** | **0.03** | **0.06** |
| ***Ruminiclostridium 9, %*** | **1.4 [0.4]** | **1.4 [0.6]** | **0.76** | **0.83** |
| ***Ruminiclostridium*, %** | **1.2 [1.1]** | **1.0 [0.8]** | **0.32** | **0.44** |
| ***Akkermansia*, %** | **0.1 [0.4]** | **0.6 [2.0]** | **0.03** | **0.06** |
| ***Rikenellaceae RC9 gut group*, %** | **0.8 [0.6]** | **0.7 [0.6]** | **0.45** | **0.56** |
| ***Muribaculum*, %** | **0.8 [0.7]** | **0.6 [0.5]** | **0.03** | **0.06** |
| ***Blautia*, %** | **0.3 [0.8]** | **0.6 [0.9]** | **0.08** | **0.16** |
| ***Alloprevotella*, %** | **0 [0]** | **0 [0]** | **0.18** | **0.27** |

Values expressed as median [interquartile range]. *P-values obtained using the Wilcoxon rank-sum test; ^†^Adjusted P-values were determined using the Benjamini-Hochberg method.
