# Supplementary material for: Effects of sex and chronic cigarette smoke exposure on the mouse cecal microbiome
Source: PLoS One. 2020 Apr 6;15(4):e0230932. doi: 10.1371/journal.pone.0230932 (PMC7135149; doi:10.1371/journal.pone.0230932)
Supplement: S6 Table — (DOCX) [file pone.0230932.s012.docx]

**S6 Table.** **Comparisons of different alpha diversity metrics between male, female and ovariectomized female groups.**

| **Metric** | **Males**  **(n=20)**  **(n=** | **Females**  **(n=20)** | **Ovariectomized Females**  **(n=18)** | **P-value*** |
| --- | --- | --- | --- | --- |
| **Richness** | **166 [30]** | **176 [33]** | **185 [26]** | **0.19** |
| **Shannon Index** | **5.6 [0.4]** | **6.0 [0.3]** | **5.6 [0.4]** | **< 0.001** |
| **Pairwise Comparisons:**^†^ | | | | |
| **Females vs. Ovariectomized Females: adj. p=0.003** | | | | |
| **Females vs. Males: adj. p=0.002** | | | | |
| **Males vs. Ovariectomized Females: adj. p=0.84** | | | | |
| **Evenness** | **0.76 [0.07]** | **0.80 [0.03]** | **0.74 [0.02]** | **0.001** |
| **Pairwise Comparisons:**^†^ | | | | |
| **Females vs. Ovariectomized Females: adj. p=0.002** | | | | |
| **Females vs. Males: adj. p=0.004** | | | | |
| **Males vs. Ovariectomized Females: adj. p=0.66** | | | | |
| **Faith’s PD** | **12.4 [0.8]** | **12.5 [0.7]** | **12.4 [1.0]** | **0.89** |

Values expressed as median [interquartile range]. *P-values obtained using the Kruskal-Wallis test; ^†^Adjusted P-values were determined using the Benjamini-Hochberg method.
