# Supplementary material for: Effects of sex and chronic cigarette smoke exposure on the mouse cecal microbiome
Source: PLoS One. 2020 Apr 6;15(4):e0230932. doi: 10.1371/journal.pone.0230932 (PMC7135149; doi:10.1371/journal.pone.0230932)
Supplement: S7 Table — (DOCX) [file pone.0230932.s013.docx]

**S7 Table.** **Comparisons of different alpha diversity metrics after stratification by smoke exposure and sex.**

| **Metric** | **CF**  **(n=10)** | **CM**  **(n=10)** | **COF**  **(n=10)** | **SF**  **(n=10)** | **SM**  **(n=10)** | **SOF**  **(n=8)** | **P-value*** |
| --- | --- | --- | --- | --- | --- | --- | --- |
| **Richness** | **174 [22]** | **178 [35]** | **186 [26]** | **178 [33]** | **162 [22]** | **175 [34]** | **0.40**  **4** |
| **Shannon Index** | **5.9 [0.4]** | **5.7 [0.3]** | **5.7 [0.5]** | **6.0 [0.1]** | **5.4 [0.5]** | **5.5 [0.3]** | **0.005** |
| **Pairwise Comparisons:**^†^ | | | | | | | |
| **CF vs SM: adj. p=0.03** | | | | | | | |
| **SF vs SM: adj. p=0.02** | | | | | | | |
| **SF vs SOF: adj. p=0.03** | | | | | | | |
| **All other comparisons: adj. p>0.05** | | | | | | | |
| **Evenness** | **0.80 [0.04]** | **0.78 [0.06]** | **0.75 [0.02]** | **0.80 [0.01]** | **0.74 [0.06]** | **0.74 [0.03]** | **0.004** |
| **Pairwise Comparisons:**^†^ | | | | | | | |
| **CF vs SM: adj. p=0.02** | | | | | | | |
| **CF vs SOF: adj. p=0.03** | | | | | | | |
| **SF vs SM: adj. p=0.03** | | | | | | | |
| **SF vs SOF: adj. p=0.03** | | | | | | | |
| **All other comparisons: adj. p>0.05** | | | | | | | |
| **Faith’s PD** | **12.4 [1.2]** | **12.4 [1.1]** | **12.2 [0.8]** | **12.5 [0.9]** | **12.5 [0.7]** | **12.7 [1.7]** | **0.81** |

Values expressed as median [interquartile range]. P-values obtained using the Kruskal-Wallis test. ^†^Adjusted P-values were determined using the Benjamini-Hochberg method. Legend: CF = control female, CM = control male, COF = ovariectomized control female, SF = smoke-exposed female, SM = smoke-exposed male, and SOF = ovariectomized smoke-exposed female.
