# Supplementary material for: Effects of sex and chronic cigarette smoke exposure on the mouse cecal microbiome
Source: PLoS One. 2020 Apr 6;15(4):e0230932. doi: 10.1371/journal.pone.0230932 (PMC7135149; doi:10.1371/journal.pone.0230932)
Supplement: S8 Table — (DOCX) [file pone.0230932.s014.docx]

**S8 Table. Multiple pairwise comparisons related to beta diversity analyses after stratification by smoke exposure and sex**

| **Group 1** | **Group 2** | **P-value*** | **Adjusted**  **P-value^†^** |
| --- | --- | --- | --- |
| **CF** | **CM** | **0.007** | **0.008** |
| **CF** | **COF** | **0.001** | **0.002** |
| **CF** | **SF** | **0.003** | **0.004** |
| **CF** | **SM** | **0.001** | **0.002** |
| **CF** | **SOF** | **0.001** | **0.002** |
| **CM** | **COF** | **0.102** | **0.10** |
| **CM** | **SF** | **0.001** | **0.002** |
| **CM** | **SM** | **0.001** | **0.002** |
| **CM** | **SOF** | **0.001** | **0.002** |
| **COF** | **SF** | **0.001** | **0.002** |
| **COF** | **SM** | **0.001** | **0.002** |
| **COF** | **SOF** | **0.001** | **0.002** |
| **SF** | **SM** | **0.001** | **0.002** |
| **SF** | **SOF** | **0.002** | **0.003** |
| **SM** | **SOF** | **0.01** | **0.01** |

Legend: smoke-exposed female (SF, n=10), smoke-exposed male (SM, n=10), ovariectomized smoke-exposed female (SOF, n=8), control female (CF, n=10), control male (CM, n=10), and ovariectomized control female (COF, n=10). *P-values obtained using the Kruskal–Wallis test; ^†^Adjusted P-values were determined using the Benjamini-Hochberg method.
