# Supplementary material for: Effects of sex and chronic cigarette smoke exposure on the mouse cecal microbiome
Source: PLoS One. 2020 Apr 6;15(4):e0230932. doi: 10.1371/journal.pone.0230932 (PMC7135149; doi:10.1371/journal.pone.0230932)
Supplement: S9 Table — (DOCX) [file pone.0230932.s015.docx]

**S9 Table.** **Relative taxa abundance comparisons at the phylum level between male, female and ovariectomized female groups.**

| **Metric** | **Males**  **(n=20)**  **(n=** | **Females**  **(n=20)** | **Ovariectomized Females**  **(n=18)** | **P-value*** | **Adjusted**  **P-value**^†^ |
| --- | --- | --- | --- | --- | --- |
| ***Bacteroidetes, %*** | **57.5**  **[6.7]** | **57.8**  **[12.6]** | **62.4**  **[12.3]** | **0.23** | **0.26** |
| ***Firmicutes, %*** | **35.1**  **[5.5]** | **35.2**  **[12.1]** | **31.3**  **[10.1]** | **0.15** | **0.26** |
| ***Epsilonbacteraeota*, %** | **2.0**  **[2.5]** | **3.6**  **[2.1]** | **3.0**  **[4.7]** | **0.26** | **0.26** |

Values expressed as median [interquartile range]. * P-values obtained using the Kruskal-Wallis test. ^†^Adjusted P-values were determined using the Benjamini-Hochberg method.
