# Supplementary material for: Effects of sex and chronic cigarette smoke exposure on the mouse cecal microbiome
Source: PLoS One. 2020 Apr 6;15(4):e0230932. doi: 10.1371/journal.pone.0230932 (PMC7135149; doi:10.1371/journal.pone.0230932)
Supplement: S10 Table — (DOCX) [file pone.0230932.s016.docx]

**S10 Table.** **Relative taxa abundance comparisons at the phylum level after stratification by smoke exposure and sex.**

| **Phylum** | **CF**  **(n=10)** | **CM**  **(n=10)** | **COF**  **(n=10)** | **SF**  **(n=10)** | **SM**  **(n=10)** | **SOF**  **(n=8)** | **P-value*** | **Adjusted**  **P-value**^†^ |
| --- | --- | --- | --- | --- | --- | --- | --- | --- |
| ***Bacteroidetes*, %** | **57.9**  **[13.9]** | **59.5**  **[11.3]** | **64.7**  **[12.9]** | **57.3**  **[8.1]** | **55.8**  **[4.0]** | **60.4**  **[10.7]** | **0.49** | **0.57** |
| ***Firmicutes*, %** | **34.2**  **[13.4]** | **34.5**  **[11.0]** | **30.9**  **[12.1]** | **36.7**  **[7.9]** | **35.3**  **[1.9]** | **31.3**  **[8.3]** | **0.57** | **0.57** |
| ***Epsilonbacteraeota*, %** | **4.2**  **[2.9]** | **1.6**  **[1.7]** | **0.0**  **[3.2]** | **2.6**  **[2.1]** | **3.3**  **[3.6]** | **4.2**  **[1.4]** | **0.02** | **0.06** |

Values expressed as median [interquartile range]. *P-values obtained using the Kruskal–Wallis test; ^†^Adjusted P-values were determined using the Benjamini-Hochberg method. Legend: CF = control female, CM = control male, COF = ovariectomized control female, SF = smoke-exposed female, SM = smoke-exposed male, and SOF = ovariectomized smoke-exposed female.
