# Supplementary material for: Effects of sex and chronic cigarette smoke exposure on the mouse cecal microbiome
Source: PLoS One. 2020 Apr 6;15(4):e0230932. doi: 10.1371/journal.pone.0230932 (PMC7135149; doi:10.1371/journal.pone.0230932)
Supplement: S11 Table — (DOCX) [file pone.0230932.s017.docx]

**S11 Table.** **Relative taxa abundance comparisons at the genus level between male, female and ovariectomized female groups.**

| **Genus*** | **Males**  **(n=20)**  **(n=** | **Females**  **(n=20)** | **Ovariectomized Females**  **(n=18)** | **P-value*** | **Adjusted**  **P-value**^†^ |
| --- | --- | --- | --- | --- | --- |
| ***Prevotellaceae UCG001*, %** | **20.7**  **[9.7]** | **11.6**  **[7.2]** | **19.7**  **[7.4]** | **0.001** | **0.01** |
| ***Lachnospiraceae***  ***NK4A136 group*, %** | **15.7**  **[5.4]** | **13.3**  **[3.8]** | **9.8**  **[6.0]** | **0.004** | **0.02** |
| ***Alistipes*, %** | **4.1**  **[5.1]** | **4.3**  **[2.3]** | **3.5**  **[5.9]** | **0.93** | **0.93** |
| ***Prevotellaceae***  ***NK3B31group*, %** | **5.5**  **[5.2]** | **2.7**  **[4.0]** | **5.7**  **[5.7]** | **0.45** | **0.64** |
| ***Bacteroides*, %** | **3.4**  **[2.2]** | **5.7**  **[4.1]** | **4.1**  **[4.1]** | **0.08** | **0.20** |
| ***Helicobacter*, %** | **2.0**  **[2.5]** | **3.6**  **[2.1]** | **3.0**  **[4.7]** | **0.25** | **0.48** |
| ***Uncultured***  ***Bacteroidales bacterium*, %** | **0.8**  **[0.9]** | **2.8**  **[2.5]** | **2.3**  **[2.8]** | **<0.001** | **0.005** |
| ***Oscillibacter*, %** | **2.1**  **[1.8]** | **2.0**  **[1.3]** | **2.2**  **[1.6]** | **0.53** | **0.64** |
| ***Ruminiclostridium 9, %*** | **1.4**  **[0.4]** | **1.2**  **[0.7]** | **1.5**  **[0.6]** | **0.56** | **0.64** |
| ***Ruminiclostridium*, %** | **0.8**  **[0.6]** | **1.5**  **[1.0]** | **1.0**  **[0.8]** | **0.013** | **0.039** |
| ***Akkermansia*, %** | **0.2**  **[2.1]** | **0.3**  **[0.8]** | **0.06**  **[0.5]** | **0.49** | **0.64** |
| ***Rikenellaceae***  ***RC9 gut group*, %** | **0.8**  **[0.5]** | **0.8**  **[0.4]** | **0.7**  **[0.9]** | **0.75** | **0.80** |
| ***Muribaculum*, %** | **0.5**  **[0.4]** | **1.1**  **[0.8]** | **0.6**  **[0.4]** | **0.009** | **0.03** |
| ***Blautia*, %** | **0.4**  **[0.9]** | **0.6**  **[1.3]** | **0.4**  **[0.7]** | **0.51** | **0.64** |
| ***Alloprevotella*, %** | **0.0**  **[0.0]** | **0.0**  **[0.0]** | **0.0**  **[0.0]** | **0.10** | **0.22** |

Values expressed as median [interquartile range]. *P-values obtained using the Kruskal–Wallis test; ^†^Adjusted P-values were determined using the Benjamini-Hochberg method.
