# Supplementary material for: Effects of sex and chronic cigarette smoke exposure on the mouse cecal microbiome
Source: PLoS One. 2020 Apr 6;15(4):e0230932. doi: 10.1371/journal.pone.0230932 (PMC7135149; doi:10.1371/journal.pone.0230932)
Supplement: S12 Table — (DOCX) [file pone.0230932.s018.docx]

**S12 Table.** **Relative taxa abundance comparisons at the genus level after stratification by smoke exposure and sex.**

| **Genus*** | **CF**  **(n=10)** | **CM**  **(n=10)** | **COF**  **(n=10)** | **SF**  **(n=10)** | **SM**  **(n=10)** | **SOF**  **(n=8)** | **P-value*** | **Adjusted**  **P-value**^†^ |
| --- | --- | --- | --- | --- | --- | --- | --- | --- |
| ***Prevotellaceae UCG001*, %** | **12.2**  **[5.6]** | **23.1**  **[9.8]** | **18.6**  **[5.9]** | **11.4**  **[7.8]** | **20.4**  **[7.3]** | **20.2**  **[6.1]** | **0.018** | **0.04** |
| ***Lachnospiraceae***  ***NK4A136 group*, %** | **13.1**  **[4.2]** | **14.7**  **[2.4]** | **10.2**  **[5.8]** | **13.3**  **[4.7]** | **16.5**  **[6.7]** | **9.0**  **[6.4]** | **0.034** | **0.056** |
| ***Alistipes*, %** | **5.5**  **[3.9]** | **7.8**  **[3.8]** | **8.1**  **[3.8]** | **3.3**  **[1.6]** | **2.6**  **[1.4]** | **2.4**  **[0.7]** | **<0.001** | **<0.001** |
| ***Prevotellaceae***  ***NK3B31group*, %** | **2.7**  **[3.4]** | **2.4**  **[2.6]** | **3.3**  **[4.9]** | **2.7**  **[5.0]** | **6.8**  **[2.6]** | **8.4**  **[2.9]** | **0.006** | **0.02** |
| ***Bacteroides*, %** | **4.1**  **[4.5]** | **2.6**  **[1.7]** | **2.8**  **[2.7]** | **6.3**  **[1.6]** | **3.8**  **[1.8]** | **5.1**  **[2.5]** | **0.03** | **0.056** |
| ***Helicobacter*, %** | **4.1**  **[2.9]** | **1.6**  **[1.7]** | **0.0**  **[3.2]** | **2.6**  **[2.1]** | **3.3**  **[3.6]** | **4.2**  **[1.4]** | **0.019** | **0.04** |
| ***Uncultured***  ***Bacteroidales bacterium*, %** | **2.5**  **[2.1]** | **1.5**  **[1.5]** | **3.7**  **[2.7]** | **3.0**  **[2.5]** | **0.5**  **[0.5]** | **1.1**  **[0.8]** | **<0.001** | **<0.001** |
| ***Oscillibacter*, %** | **1.8**  **[0.7]** | **1.8**  **[0.7]** | **2.1**  **[1.6]** | **2.0**  **[1.4]** | **3.4**  **[2.8]** | **2.4**  **[1.6]** | **0.20** | **0.25** |
| ***Ruminiclostridium 9, %*** | **1.3**  **[0.4]** | **1.3**  **[0.4]** | **1.5**  **[0.6]** | **1.1**  **[1.1]** | **1.4**  **[0.2]** | **1.4**  **[0.5]** | **0.66** | **0.66** |
| ***Ruminiclostridium*, %** | **1.8**  **[1.0]** | **0.9**  **[0.5]** | **1.1**  **[0.8]** | **1.4**  **[0.7]** | **0.8**  **[0.6]** | **0.7**  **[0.8]** | **0.06** | **0.09** |
| ***Akkermansia*, %** | **0.5**  **[0.7]** | **0.02**  **[0.2]** | **0.0**  **[0.1]** | **0.03**  **[0.6]** | **2.5**  **[3.1]** | **0.8**  **[1.4]** | **0.002** | **0.01** |
| ***Rikenellaceae***  ***RC9 gut group*, %** | **0.9**  **[0.6]** | **0.7**  **[0.4]** | **0.9**  **[1.5]** | **0.6**  **[0.4]** | **0.9**  **[0.6]** | **0.6**  **[0.6]** | **0.32** | **0.34** |
| ***Muribaculum*, %** | **1.0**  **[0.6]** | **0.6**  **[0.5]** | **0.8**  **[0.4]** | **1.1**  **[1.1]** | **0.4**  **[0.4]** | **0.5**  **[0.3]** | **0.012** | **0.04** |
| ***Blautia*, %** | **0.6**  **[0.9]** | **0.5**  **[0.7]** | **0.2**  **[0.3]** | **0.8**  **[2.1]** | **0.4**  **[1.0]** | **0.6**  **[1.1]** | **0.22** | **0.25** |
| ***Alloprevotella*, %** | **0.0**  **[0.0]** | **0.0**  **[0.0]** | **0.0**  **[0.0]** | **0.0**  **[0.0]** | **0.0**  **[0.0]** | **0.0**  **[0.0]** | **0.08** | **0.11** |

Value expressed as median [interquartile range]. *P-values obtained using the Kruskal–Wallis test; ^†^Adjusted P-values were determined using the Benjamini-Hochberg method. Legend: CF = control female, CM = control male, COF = ovariectomized control female, SF = smoke-exposed female, SM = smoke-exposed male, and SOF = ovariectomized smoke-exposed female.
